# Antitumor Anthraquinones from an Easter Island Sea Anemone: Animal or Bacterial Origin?

**DOI:** 10.3390/md17030154

**Published:** 2019-03-05

**Authors:** Ignacio Sottorff, Sven Künzel, Jutta Wiese, Matthias Lipfert, Nils Preußke, Frank D. Sönnichsen, Johannes F. Imhoff

**Affiliations:** 1 GEOMAR Helmholtz Centre for Ocean Research Kiel, Marine Microbiology, 24105 Kiel, Germany; isottorff@geomar.de (I.S.); jwiese@geomar.de (J.W.); 2 Facultad de Ciencias Naturales y Oceanográficas, Universidad de Concepción, 4070386 Concepción, Chile; 3 Max Planck Institute for Evolutionary Biology, 24306 Plön, Germany; kuenzel@evolbio.mpg.de; 4 Otto Diels Institute for Organic Chemistry, University of Kiel, 24118 Kiel, Germany; mlipfert@oc.uni-kiel.de (M.L.); npreusske@oc.uni-kiel.de (N.P.); fsoennichsen@oc.uni-kiel.de (F.D.S.)

**Keywords:** Easter Island, Actinobacteria, anthraquinones, symbionts, sea anemone, marine invertebrates, spectroscopy, chromatography

## Abstract

The presence of two known anthraquinones, Lupinacidin A and Galvaquinone B, which have antitumor activity, has been identified in the sea anemone (*Gyractis sesere*) from Easter Island. So far, these anthraquinones have been characterized from terrestrial and marine Actinobacteria only. In order to identify the anthraquinones producer, we isolated Actinobacteria associated with the sea anemone and obtained representatives of seven actinobacterial genera. Studies of cultures of these bacteria by HPLC, NMR, and HRLCMS analyses showed that the producer of Lupinacidin A and Galvaquinone B indeed was one of the isolated Actinobacteria. The producer strain, SN26_14.1, was identified as a representative of the genus *Verrucosispora*. Genome analysis supported the biosynthetic potential to the production of these compounds by this strain. This study adds *Verrucosispora* as a new genus to the anthraquinone producers, in addition to well-known species of *Streptomyces* and *Micromonospora*. By a cultivation-based approach, the responsibility of symbionts of a marine invertebrate for the production of complex natural products found within the animal’s extracts could be demonstrated. This finding re-opens the debate about the producers of secondary metabolites in sea animals. Finally, it provides valuable information about the chemistry of bacteria harbored in the geographically-isolated and almost unstudied, Easter Island.

## 1. Introduction

Since the discovery of Easter Island, compelling explorations have characterized the flora [1] and fauna [2,3,4] of this geographically isolated location. However, little has been done to understand the chemistry harbored in this territory. The best-known finding is the discovery of rapamycin in a soil dwelling actinobacterial representative, which serves as an immunosuppressive drug [5,6]. Beyond that, little progress has been made in exploiting the chemical diversity harbored by marine invertebrates and microorganisms dwelling in this territory, despite the high degree of endemism found [7]. 

Marine invertebrates are immensely diverse, well distributed in the world oceans [8], and widely known to contain medicinally relevant molecules [9,10,11]. While these metabolites play different roles in nature, e.g., they act as chemical defense, chemical communication or reproductive signaling molecules, they also find application as human medicines [12]. During the last decades, much effort has been made to identify and characterize the chemicals contained in marine invertebrates. This has resulted in the discovery of astonishing chemicals with novel biological activities and chemical scaffolds [12,13,14]. 

Further, the immense progress in DNA sequencing technologies, the development of bioinformatics, and an improvement in analytical techniques enables the identification of the source of the chemicals. Thus, several molecules contained in marine invertebrates have now been shown to actually be of microbial origin [15,16]. It is expected that with the increasing availability of metagenomic information more identifications of the real producers of these metabolites will be made and will establish the metabolite relevance for the interaction of host, symbiont, and the environment. 

Marine sponges represent a classic example of marine invertebrates that harbor microbes producing secondary metabolites. They have been studied in detail to determine the origin of the metabolites [17,18]. Another example is the producer of the approved anticancer drug Yondelis (Ecteinascidin-743). This compound was first assigned to the tunicate *Ecteinascidia turbinata*, but later identified as the product of a microbe, Candidatus *Endoecteinascidia frumentensis* [16]. 

Anthraquinones have been characterized in different marine invertebrates, for example crinoids [19] and sponges [20]. Anthraquinones have broad biological activity and are substances of pharmaceutical relevance for revealing antitumor [21], antibacterial [22], antifungal [23] and epigenetic modulator activities [24]. Two recently isolated anthraquinones, Lupinacidin A (**1**) and Galvaquinone B (**2**), have been reported to be produced by Actinobacteria belonging to the genera *Streptomyces* and *Micromonospora*. Lupinacidin A (**1**) was firstly reported as a specific inhibitor on murine colon 26-L5 carcinoma cells [25], and Galvaquinone B (**2**) showed moderate cytotoxicity against non-small-cell lung cancer cells Calu-3 and H2887, in addition of epigenetic modulatory activity [24].

Herein, we report the identification of two known anthraquinone molecules, Lupinacidin A (**1**) and Galvaquinone B (**2**), contained in an Easter Island sea anemone, *Gyractis sesere*. Interestingly, so far these molecules have been only characterized from microbial origin. Therefore, we undertook the isolation and culture of the Actinobacteria associated with this marine invertebrate. The culture, chemical and genomic evaluation of these bacteria showed that the real producer of the metabolites was an Actinobacterium belonging to the genus *Verrucosispora* and not the sea anemone.

## 2. Results and Discussion

### 2.1. Sea Anemone Dereplication

**Figure 1:**
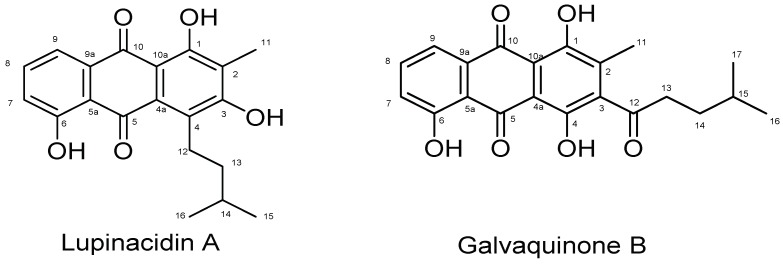
Identified molecules from the Easter Island sea anemone *Gyractis sesere*.

Samples of the sea anemone *Gyractis sesere* [26], also known as *Actiniogeton rapanuiensis* [27], were collected in the intertidal zone of Easter Island, South Pacific Ocean, and extracted with chloroform to give 8 mg of a yellowish residue. The HRLCMS analysis of this crude extract showed the presence of two previously identified antitumor anthraquinones (Figure 1) [24,25], Lupinacidin A (**1**) ([M + H]^+^ m/z 341.1378) and Galvaquinone B (**2**) ([M + H]^+^ m/z 369.3510), which were confirmed by complete NMR-spectroscopic characterization. 

**Figure 2:**
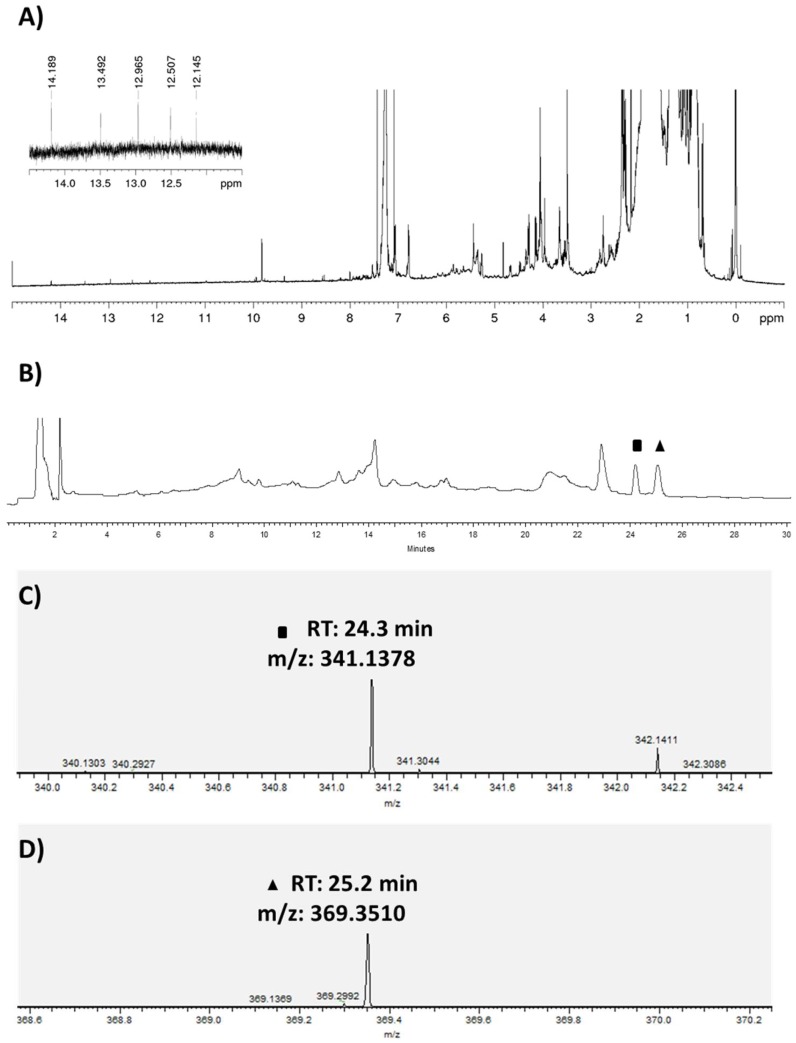
Chemical analysis of the crude extract of the sea anemone *Gyractis sesere*. (**A**) ^1^H NMR spectra of the crude extract of marine anemone *Gyractis sesere* acquired in CDCl_3_, 600 MHz. Highlighted in the zoomed area are the frequencies of characteristic resonances originating from hydroxyl exchangeable protons in vicinity to ketogroups. (**B**) UV chromatogram (254 nm) of the crude extract of the sea anemone *Gyractis sesere* highlighting the specific peaks for ■RT: 24.3 min, and ▲: RT: 25.2 min. (**C**) High resolution mass for ■ (m/z [M + H]^+^ 341.1378) and (**D**) high resolution mass for ▲ (2) (m/z [M + H]^+^ 369.3510). *RT: Retention Time.

Interestingly, the crude extract of the sea anemone (Figure 2) shows five resonances above 10 ppm; a region which is characteristic for hydroxyl protons. The resonances at δ 14.18 and 12.96 ppm are assigned to the groups at C-1 and C-6 of Lupinacidin A (**1**), as their vicinity and consequential hydrogen bonding to the ketogroups slows down their exchange. Similarly, the resonances at δ 13.49, 12.50, and 12.14 ppm originate from the three hydroxylprotons in Galvaquinone B (**2**).

Other main peaks found in the sea anemone crude extract were peaks at RT 14.2 min with a HRMS [M + H]^+^ m/z 295.19009 and at RT 23 min with a HRMS [M + H]^+^ m/z 256.26312. Both exact masses were evaluated using the MarinLit database, however their HRMS did not match any known compound to-date. 

### 2.2. Bacterial Metabolites and Harbored Bacteria

**Figure 3:**
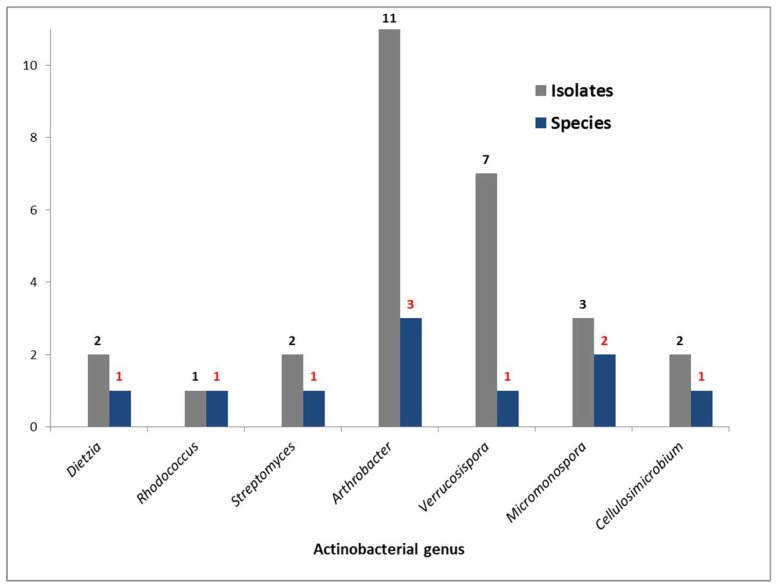
Genera and number of Actinobacteria species strains isolated from the sea anemone *Gyractis sesere*.

Lupinacidin A (**1**) and Galvaquinone B (**2**) have so far only been characterized in actinobacterial representatives, specifically from the genera *Streptomyces* [24,28] and *Micromonospora* [25], raising the question of the origin of these compounds in the sea anemone extract. Thus, we cultivated the Actinobacteria harbored by this sea anemone to determine if the anthraquinone producer was a bacterium or the sea anemone. Isolation media and the respective obtained strains are specified in Supplementary Table S1. Ten strains were identified through analysis of the 16S rRNA gene sequences as members of the genera *Micromonospora*, *Streptomyces*, *Verrucosispora*, *Dietzia*, *Arthrobacter*, *Rhodococcus*, and *Cellulosimicrobium* (Figure 3). Remarkably, *Gyractis sesere* harbors a high number of Actinobacteria genera, in total seven; the most abundant genus being *Micromonospora* with three different species, followed by *Arthrobacter* with two different species. Other actinobacterial genera were present with only one species each. Outstandingly, the only isolate belonging to the *Verrucosispora* genus was the most abundant single Actinobacterium in the sea anemone. 

### 2.3. Bacterial Growth

To evaluate the production of the anthraquinones by the isolated bacteria, we selected the actinobacterial representatives for growth experiments that were most closely related to known producers of the genera *Streptomyces*, *Micromonospora* and *Verrucosispora*. In addition, we also grew the *Dietzia* representative due to the lack of comprehensive information about its secondary metabolite production. On the other hand, *Rhodococcus*, *Arthrobacter*, and *Cellulosimicrobium* were omitted here because of their known poor production of secondary metabolites.

**Figure 4:**
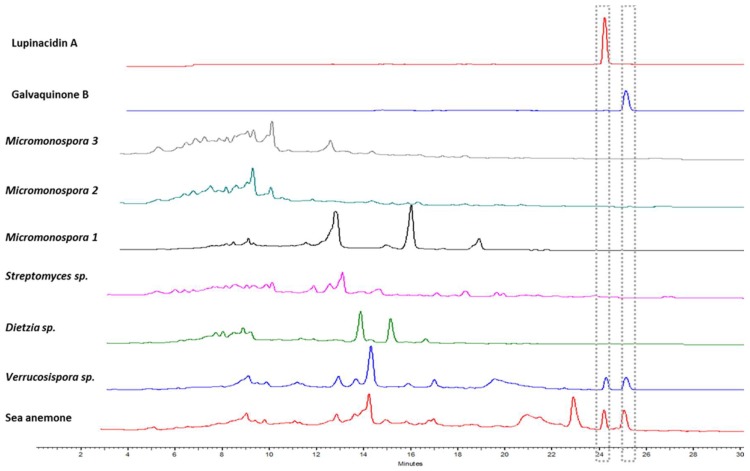
HPLC chromatograms of the crude extracts of the sea anemone *Gyractis sesere*, its respective actinobacterial isolates, and the purified anthraquinones, Lupinacidin A (**1**) and Galvaquinone B (**2**). Approximate retention times of Lupinacidin A (**1**) and Galvaquinone B (**2**) are highlighted by boxes.

The growth yield of the selected Actinobacteria was in the range of 20 to 100 mg crude extract. The chromatograms of HPLC analyses of the crude extracts were compared in order to facilitate the metabolic comparison between grown bacteria, the sea anemone and the pure substances (Figure 4).

By comparison of chromatograms and retention times, we observed that Lupinacidin A (**1**) and Galvaquinone B (**2**) were only present in the crude extract of the sea anemone *Gyractis sesere* and *Verrucosispora sp*. SN26_14.1, and not in the other actinobacterial representatives. Clearly, *Verrucosispora* must be the producer of the anthraquinones. Further, it is obvious that the metabolites of *Verrucosispora sp.* strain SN26_14.1 are dominant in the marine invertebrate. The chromatograms of *Verrucosispora* and sea anemone extracts are nearly identical and differ only slightly in the region of retention time 20–23 min. Notably the chromatogram of the sea anemone extract also does not show any peaks that suggest the presence of metabolites of any other of the cultivated bacteria. Together, this strongly suggests *Verrucosispora* appeared to be the most abundant microbe in the sea anemone biomass during the collection. 

The subtle difference in the metabolite profiles between *Verrucosispora* and sea anemone extract in the retention time region 20–23 min appears to be to metabolites produced by the sea anemone itself. Overall, the amount appears to be surprisingly small. This may however be caused by the isolation methodology (chloroform extraction), that prioritizes lipophilic substances and selects against the isolation of polar compounds such as peptides. 

### 2.4. Actinobacterial Producer

To confirm and replicate the production of these metabolites, we undertook a scale up culture of *Verrucosispora* sp. SN26_14.1. Thus, 10 L of the Actinobacterium culture were grown, and extracted through the use of amberlite XAD-16 resin, yielding 1 g of crude extract with a brownish coloration. This extract was subjected to stepwise flash chromatography using iso-octane and ethyl acetate gradients, which produced a total of ten fractions. The fractions were evaluated through HPLC to find the fractions containing Lupinacidin A (**1**) and Galvaquinone B (**2**). The chromatogram evaluation showed that only the orange colored fraction two, which was eluted with 90% iso-octane and 10% ethyl acetate, contained 78 mg of metabolites enriched with Lupinacidin A (**1**) and Galvaquinone B (**2**). The purification of Lupinacidin A (**1**) and Galvaquinone B (**2**) was achieved through HPLC using normal and reverse phase chromatography.

Lupinacidin A (**1**) was isolated as a yellow powder, with a yield of 11 mg from a 10 L culture, which suggested to be an intermediate yield compared with *Streptomyces* and *Micromonospora* producers [24,25,28]. High resolution APCI-MS gave an [M + H]^+^ adduct of m/z 341.1378, which results in a molecular formula of C_20_H_20_O_5_. The calculation of the degree of unsaturation indicated 11 degrees. ^1^H NMR showed the characteristic exchangeable protons of (**1**) at δ at 14.18 and 12.96 ppm, in addition to the three neighboring aromatic proton signals δ 7.26, 7.62, and 7.79 ppm that showed the expected coupling pattern for three neighboring aromatic protons in a para-ortho, ortho-meta relationship (two duplets, and one duplet of duplets). The ^13^C NMR experiment showed 20 carbons of which two represented ketone signals (δ 190.2 and 186.9 ppm), 12 aromatic carbons, and six aliphatic carbons (see Table 1). Two-dimensional NMR experiments, Homonuclear COrrelated SpectroscopY (COSY), Heteronuclear Single Quantum Correlation (HSQC), and Heteronuclear Multiple Bond Correlation (HMBC), helped to confirm the identity of the molecules. These data were in agreement with the published information [24,25,28].

Galvaquinone B (**2**) was isolated as a red powder, with a yield of 7 mg from a 10 L culture, which is an intermediate yield compared with *Streptomyces* and *Micromonospora* producers [24,28]. High resolution APCI-MS gave an [M + H]^+^ adduct of m/z 369.3510 and a molecular formula of C_21_H_20_O_6_. The calculation of the degree of unsaturation indicated 12 degrees. Galvaquinone B (**2**) showed characteristic exchangeable proton signals at δ 13.49, 12.50, and 12.14 ppm, respectively. Aromatic signals were similar to those found in compound (**1**), showing three neighboring aromatic protons in a para-ortho, ortho-meta relationship (two duplets, and one duplet of duplets), but with a higher frequency (see Table 1). The ^13^C NMR experiment showed 21 carbons of which three represented ketone signals (δ 205, 190.2 and 186.9 ppm), 12 aromatic carbons, and six aliphatic carbons (see Table 1). Two dimensional experiments (COSY, HSQC, HMBC) confirmed the identity of the molecules and were in agreement with the published data [24,28].

### 2.5. Phylogeny of the Producer

**Figure 5:**
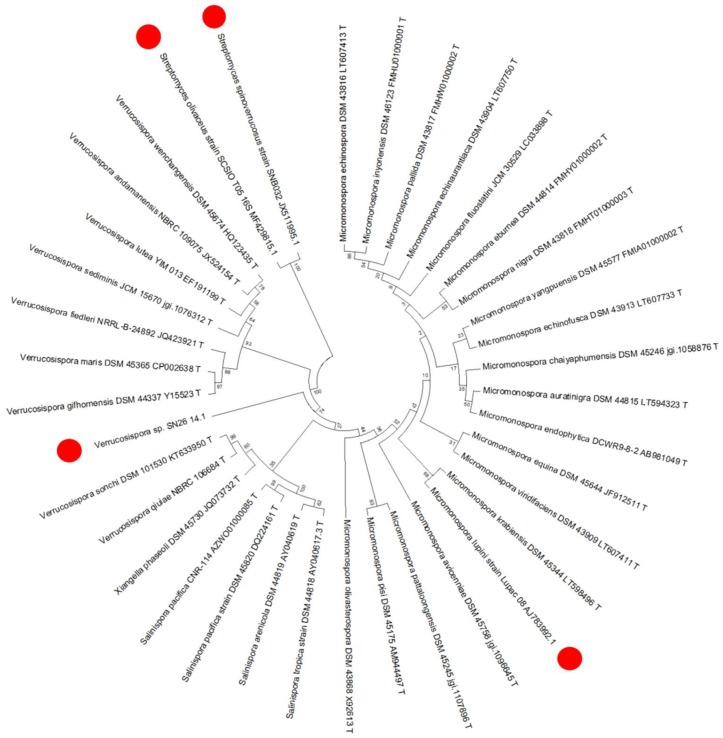
Phylogenetic tree based on 16S rRNA gene sequence of *Verrucosispora sp*. SN26_14.1. The tree was calculated using a neighbor-joining statistical method and Jukes–Cantor model. • Red dots highlight Lupinacidin A (**1**) and Galvaquinone B producers (**2**).

To determine whether the present isolate was a new species and to evaluate its evolutionary relationship, we performed a phylogenetic evaluation of the strain based of the 16S rRNA gene sequence (Figure 5). The evaluation of the 16S gene showed a high similarity (99%) to the next related type strain, *Verrucosispora maris* DSM 45365^T^. However, analysis of the gyrase subunit B taxonomic marker (gyrB) showed 94.4% similarity to *Verrucosispora maris* DSM 45365^T^. The construction of the phylogenetic tree with the closest relatives in terms of the 16S rRNA gene sequence as well as known producers of (**1**) and (**2**) confirmed that the producer strain SN26_14.1 belongs to the genus *Verrucosispora*, and that quite likely it represents a new species within the genus. This result represents the first report of anthraquinone production for the *Verrucosispora* genus. Interestingly, it appears that anthraquinones are more widespread metabolites in the *Actinobacteria* phylum, since compounds (**1**) and (**2**) have now been found in three actinobacterial genera, *Micromonospora*, *Streptomyces*, and *Verrucosispora* producers [24,25,28].

### 2.6. Biosynthesis

**Figure 6:**
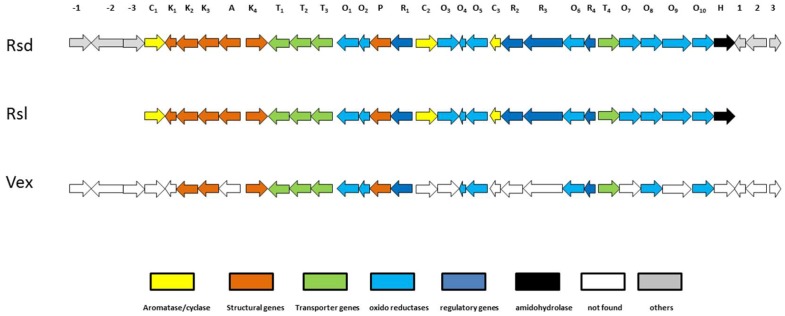
Biosynthetic gene cluster of anthraquinones producers. Rsd: *Streptomyces olivaceus* SCSIO T05 gene cluster [28], Rsl: *Streptomyces bottropensis* (*Streptomyces.* sp. Gc C4/4) gene cluster [29], Vex: *Verrucosispora* sp. SN26_14.1. **C1**: aromatase, **K_1_**: acyl carrier protein, **K_2_**: ketosynthase (beta), **K_3_**: ketosynthase (alpha), **A**: acyl transferase, **K_4_**: 3-oxoacyl-ACP synthase III, **T_1_**: ABC-transporter (substrate binding), **T_2_**: ABC-transporter (ATP binding), **T_3_**: ABC-transporter trans-membrane, **O_1_**: luciferase-like monooxygenase, **O_2_**: flavin reductase, **P**: phosphotransferase, **R_1_**: SARP family regulator, **C_2_**: second ring cyclase, **O_3_**: 3-oxoacyl-ACP reductase, **O_4_**: anthrone monooxygenase, **O_5_**: NADH: flavin oxidoreductase, **C_3_**: cyclase, **R_2_**: SARP regulatory protein, **R_3_**: LAL-family regulator, **O_6_**: luciferase-like monooxygenase, **R_4_**: MarR family transcriptional regulator, **T_4_**: drug resistance transporter, **O_7_**: putative NADPH quinone reductase, **O_8_**: putative NADPH: quinone oxidoreductase, **O_9_**: FAD-dependent oxidoreductase, **O_10_**: C9-keto reductase, **H**: amidohydrolase, **-3**: unknown function, -2: major facilitator superfamily protein, **-1**: Transcriptional regulatory protein, **1**: cupin, **2**: citrate/H^+^ symporter, **3**: transcriptional regulator.

Recently, the biosynthetic machinery for the production of compound (**1**) and (**2**) was described as a type II polyketide synthase (PKS) that features a special Baeyer−Villiger type rearrangement, and was allocated to an Rsd gene cluster in *Streptomyces olivaceus* SCSIO T05 [28]. The Rsd biosynthetic gene cluster (BGC) showed great similarity to the Rsl BGC reported for the production of rishirilide A and B in *Streptomyces bottropensis* (also known as *Streptomyces. sp*. Gç C4/4) [29]. The BGC Rsd is responsible for the production of six molecules (rishirilide B, rishirilide C, Lupinacidin A (**1**), Lupinacidin D, Galvaquinone A and Galvaquinone B (**2**)), and among them compound (**1**) and (**2**) [28]. This raised the question of whether in *Verrucosispora,* compounds (**1**) and (**2**) follow the same biochemical assembly line as described for *Streptomyces*. Thus, the genome of *Verrucosispora sp.* SN26_14.1 was sequenced using Illumina MiSeq. Although the obtained short reads were not complemented with a long read sequencing technology as PacBio, we were still able to obtain a 6.9 Mb draft genome (NCBI Bioproject Access # PRJNA522941). This data was annotated with Prokka and analyzed with the Antismash online platform [30] to identify the secondary metabolite biosynthesis gene clusters. As shown in Figure 6, the draft genome of *Verrucosispora* sp. SN26_14.1 shared 60% of the genes of the Rsd gene cluster, as well as to an important percentage of the genes of the Rsl BGC. The similarity of the found genes ranged from 49% to 81%. The genetic architecture found in Vex BGC was quite similar to that of the Rsd and Rsl BGC. Remarkably, we could not detect any cyclase/aromatase and amidohydrolase sequences in our draft genome. Likely, this relates to the incompleteness of our sequence. Finally, it appears reasonable that *Verrucosispora* sp. SN26_14.1 follows the same biosynthetic machinery for the production of Lupinacidin A (**1**) and Galvaquinone B (**2**) as found for *Streptomyces* species (Figure 6).

### 2.7. Antibiotic Activity Test

We performed a disc diffusion antibiotic test as a preliminary evaluation to determine if Lupinacidin A (**1**) and Galvaquinone B (**2**) have an inhibition effect on bacteria. As a positive control, we used streptomycin at a concentration of 25 μg/disc. The results showed that Lupinacidin A (**1**) and Galvaquinone B (**2**) did not produce any growth inhibition against the Gram-positive bacterium *Staphylococcus lentus* DSM 20352^T^, and neither against the Gram-negative bacterium *Escherichia coli* DSM 498^T^. In contrast, the positive control, streptomycin produced an inhibition halo of 22 mm for Gram-negative and 18 mm for Gram-positive bacteria.

## 3. Materials and Methods 

### 3.1. Sample Collection

The sea anemone *Gyractis sesere* (also known as *Actiniogeton rapanuiensis*) was sampled from the coastal zone of Easter Island (27°08′45.1″S, 109°25′50.0″W) by the first author (Chilean citizen), in March 2016. The sampling site was outside the Isla de Pascua national park, and the sample was taken in agreement with regulations by the Chilean government. The sample was stored at 0 °C one hour after the sampling process.

### 3.2. Sea Anemone Dereplication

10 g of the sea anemone *Gyractis sesere* (wet weight) were thawed and homogenized with a mortar and pestle. When a creamy consistency was obtained, the tissue was transferred to a 250 mL beaker and 50 mL of chloroform was added. This extraction procedure was repeated three times. The obtained chloroform extract was concentrated until dryness under reduced pressure in a rotatory evaporator. The dried extract was resuspended in 1 L deionized water and transferred to a separation funnel, where it was partitioned with chloroform (3 × 300 mL). This process produced 8 mg of crude extract with a brownish coloration. Part of the crude extract (0.5 mg) was resuspended in methanol (HPLC grade) and injected in a HPLC (Merck Hitachi LaChrom Elite, Darmstadt, Germany) and in a HRLCMS Thermo Scientific™ Q Exactive™ Hybrid-Quadrupol-Orbitrap (Bremen, Germany), positive mode, and a 30 minute gradient of H_2_O and acetonitrile supplemented with 0.1% of formic acid. The gradient developed as following: 0 min: 90% water, 10% acetonitrile, 25 min: 0% water, 100% acetonitrile, 28 min: 0% water, 100% acetonitrile, 30 min: 90% water, 10% acetonitrile. Mass spectroscopic data was evaluated with Xcalibur^®^ (Thermo Fisher Scientific, San Jose, CA, USA), and compared with online databases (MarinLit, and Scifinder), and literature. The entire remaining sample was dissolved in deuterated chloroform (Eurisotop™, Saint-Aubin, France) and analyzed by ^1^H NMR using a Bruker (Rheinstetten, Germany) Avance 600 MHz NMR spectrometer.

### 3.3. Bacterial Isolation

Approximately 1 cm^3^ (2 g) of the sea anemone *Gyractis sesere* (wet weight) were thawed and homogenized with a sterile mortar and pestle. Subsequently, the homogenized tissue was mixed with 9 mL of Ringer’s buffer ¼ strength [31] to produce a final solution of 1:10. This solution was incubated at 56 °C for 10 min with the aim of reducing the viability of non-actinobacterial microbes. After the incubation, 1 min of vortex was applied. The inoculation of the culture media was done by adding 50 μL of the dilution into 15 cm diameter Petri dishes containing the media. The inoculum was spread out on the plate with a triangular cell spreader made of glass. Finally, plates were incubated at 25 °C in darkness. Darkness was chosen as a filtering factor to eliminate potential microalgae contamination. Four different media were prepared for the isolation of Actinobacteria from the sea anemone *Gyractis sesere*. Medium SIMA1 (*Salinispora* isolation media A1) was selected from literature and slightly modified as follows: 2.5 g starch, 1 g yeast extract, 0.5 g peptone, 1 L deionized water, and 25 g Tropic Marin™ salt (Wartenberg, Germany), 15 g/L agar [32]. The other media (BCM, BTM, and BSEM) were generated for this study as follows: BCM, 3 g chitin, 0.5 g N-acetyl glucosamine, 0.2 g K_2_HPO_4_, 0.25 g KNO_3_, 0.25 g casein, 5 mL of mineral solution, 4 mL vitamin solution, 1 L deionized water, 15 g/L Tropic Marin™ salt (Wartenberg, Germany), 12 g/L Gellan gum, pH = 7.35; BTM, 1 g trehalose, 0.25 g histidine, 0,25 g proline, 0.2 g MgCl_2*_6H_2_O, 4 mL vitamin solution, 12 g/L Gellan gum, 1 L deionized water, 15 g Tropic Marin™ salt (Wartenberg, Germany), pH = 7.2; and BSEM, 0.1 g tyrosine, 0.1 g d-galactose, 4 mL vitamin solution, 5 mL mineral solution, 1 L Baltic Sea water, 16 g/L agar, pH = 7.4. Mineral salt solution contained 1 L distillated water, 50 mg FeSO_4*_7H_2_O, 50 mg ZnCl_2_, and 50 mg CuSO_4_. Vitamin solution contained 1 L distillated water, 5 mg thiamine_*_HCl, 5 mg riboflavin, 5 mg niacin, 5 mg pyrodoxine HCl, 5 mg inositol, 5 mg Ca-pantothenate, 5 mg p-amino benzoic acid, and 2.5 mg biotin. 

The media were autoclaved for 35 min at 121 °C. Subsequently, the culture media were supplemented with 50 mg/L of nalidixic acid (Sigma-Aldrich, St. Louis, MO, USA) and 100 mg/L of cycloheximide (Carl Roth GmbH, Karlsruhe, Germany) [33], and poured into petri dishes. Once the sample was inoculated onto the petri dish, they were incubated for six weeks. When bacterial colonies were visually evident, we proceeded with the purification of the bacteria until obtaining an axenic culture. The isolated bacteria were conserved using Cryobank™ (Mast Diagnostica GmbH, Reinfeld, Germany) bacterial storage system. 

### 3.4. Molecular Characterization and Phylogenetic Analysis

DNA was extracted from bacterial cells by use of a DNA isolation kit, DNeasy™ (Qiagen, Hilden, Germany), following the manufacturer instructions. Subsequently, the 16S rRNA gene sequence was amplified with PCR and the use of general bacterial primers in a concentration of 10 pmol/µL, i.e., 27f and 1492r [34], 342f and 534r [35], 1387r [36] as well as 1525r [37]. PCR reagents were obtained from GE Healthcare Illustra™ PuReTaq Ready-To-Go™ PCR Beads (GE Healthcare, Glattbrugg, Switzerland) containing DNA polymerase, MgCl_2_, and dNTPs. The PCR conditions were the same as reported by Staufenberger et al. [35]. Once the PCR amplification process was terminated, a quality check of the PCR products was performed by gel electrophoresis. The sequencing process was run at the Centre for Molecular Biology at Kiel University (IKMB). The 16S rRNA gene sequences were manually curated using Chromas pro software, version 1.7.6 (Technelysium Pty Ltd., Tewantin QLD, Australia), and saved in FASTA format. Sequences were aligned with nucleotide BLAST [38] and EZbiocloud [39]. Phylogenetic analysis involved the alignment of the sequences with related reference strains in the web platform SILVA-SINA [40]. MEGA was used to delete gap sites and to run bootstrapped phylogenetic trees using a neighbor-joining model [41].

### 3.5. Bacterial Growth for Secondary Metabolites Production

For the evaluation of the secondary metabolites production, we grew the Easter Island isolated strain *Verrucosispora* sp. SN26_14.1 in 10 × 2.5 L Thomson Ultra Yield^®^ flasks (Thomson Instrument, Oceanside, CA, USA), which contained 1 L each of a modified starch-glucose-glycerol (SGG) liquid medium [31]. The composition of the production medium was: 5 g glucose, 5 g soluble starch, 5 g glycerol, 1.25 g cornsteep powder, 2.5 g peptone, 1 g yeast extract, 1.5 CaCO_3_, and 1 L deionized water. The medium was also supplemented with 15 g/L Tropic Marin™ salt (Wartenberg, Germany). The pH was adjusted to 7.7 using 1 M HCl and NaOH. The culture was kept in orbital agitation at 240 RPM, 28 °C, for 14 days in darkness.

### 3.6. Chemical Extraction, Purification and Structure Elucidation

After the growth period, 20 g/L amberlite XAD-16 (Sigma-Aldrich, St. Louis, MO, USA) was added to each culture medium flask and mixed for one hour using orbital agitation with 120 rpm. Subsequently, the resin was separated through cheesecloth filtration [42], and the liquid was discarded. Afterwards, amberlite plus cheesecloth was mounted on a glass funnel, washed with 3 L of deionized water, and eluted with 1 L of acetone [42]. Acetone was then concentrated under reduced pressure until an aqueous residue was obtained. One liter of deionized water was added to the acetone residue, and it was brought to a separation funnel. The organic molecules were extracted using 3 x 1 L of ethyl acetate. The organic phase was concentrated under reduced pressure until dryness. 

For the evaluation of the produced metabolites, we used HPLC-DAD (Merck Hitachi LaChrom Elite, Darmstadt, Germany) and a 30 min gradient of H_2_O-acetonitrile supplemented with 0.1% of formic acid. The gradient was developed as following: 0 min: 90% water, 10% acetonitrile, 25 min: 0% water, 100% acetonitrile, 28 min: 0% water, 100% acetonitrile, 30 min: 90% water, 10% acetonitrile. The gravity SB™ C-18 column was obtained from Macherey-Nagel (Düren, Germany).

The purification of chemicals involved three different steps: 1) Flash chromatography using standard silica gel 60, pore size ~ 60 Å (Macherey-Nagel, Düren, Germany) as a stationary phase, mounted in a glass Buchner funnel (D = 70 mm, H = 180 mm). The mobile phase solvents were iso-octane and ethyl acetate. The chromatographic process was developed in a stepwise increase of polarity (10% each), starting with 100% iso-octane, and 0% of ethyl acetate, and ending in 0% iso-octane and 100% ethyl acetate, resulting in 10 different fractions. 2) The fraction that contained compound (**1**) and (**2**) was selected and worked in HPLC (Merck Hitachi LaChrom Elite, Darmstadt, Germany) using a normal phase NUCLEODUR^®^ 100-5 column (4.6 × 250 mm) from Macherey-Nagel (Düren, Germany). The method used for the purification was a combination of isocratic and gradient solvent mix, with a flow rate of 1 mL/min, where A: iso-octane, B: ethyl acetate, and C: dichloromethane/methanol (50:50). The method was developed as following: 0 min: 100% A and 0% B, 3 min: 100% A and 0% B, 5 min: 95% A and 5% B, 9 min: 95% A and 5% B, 11 min: 0% A and 100% B, 13 min: 0% A and 100% B, 14 min: 10% A, 50% B, and 40% C, 16 min: 10% A, 50% B, and 40% C, 18 min: 50% A and 50% B, 19 min: 100% A and 0% B, 21 min: 100% A and 0% B. 3) The semi-purified compounds were purified through HPLC (Merck Hitachi LaChrom Elite, Darmstadt, Germany using a reverse phase C-18 column, 10 × 250 mm (YMC, Kyoto, Japan). The method used for the purification was a combination of isocratic and gradient solvent mix, with a flow rate of 2.5 mL/min. The method was developed as following: 0 min: 90% A and 10% B, 5 min: 20% A and 80% B, 9 min: 20% A and 80% B, 13 min: 0% A and 100% B, 19 min: 0% A and 100% B, 23 min: 90% A and 10% B, 25 min: 90% A, 10% B (A. water, B: acetonitrile). 

After these purification steps, Lupinacidin A (**1**) and Galvaquinone B (**2**) were obtained with high purity to perform structural elucidation experiments. HRLCMS was performed with a Thermo Scientific™ Q Exactive™ Hybrid-Quadrupol-Orbitrap (Thermo Scientific, Bremen, Germany), positive mode, and a 30 min gradient of H_2_O and acetonitrile supplemented with 0.1% of formic acid. The gradient was developed as follows: 0 min: 90% water, 10% acetonitrile, 25 min: 0% water, 100% acetonitrile, 28 min: 0% water, 100% acetonitrile, 30 min: 90% water, 10% acetonitrile. Mass spectroscopic data was evaluated with Xcalibur^®^ (Thermo Fisher Scientific, San Jose, CA, USA), and the compared with online databases (MarinLit, and Scifinder), and literature. 

Additionally, ^1^H and ^13^C NMR and two-dimensional NMR experiments (HMBC, HSQC, COSY) were acquired to characterize the main components of crude extract, and their chemical functionality. For this, compound (**1**) and (**2**) were redissolved in CDCl_3_ (Eurisotop™, Saint-Aubin, France), and transferred to NMR tubes (178 x 5.0 mm). Experiments were acquired on a Bruker (Rheinstetten, Germany) Avance spectrometer operating at 600 MHz proton frequency equipped with a cryogenically cooled triple resonance z-gradient probe head using stand pulse sequences from the Bruker experiment library. Spectra were referenced against tetramethylsilane (Sigma-Aldrich, St. Louis, MO, USA) as internal standard.

### 3.7. Genome Sequencing

The samples were prepared with the Nextera^®^ XT DNA sample preparation kit from Illumina (Illumina, San Diego, CA, USA) following the manufacturer’s protocol. Afterwards the samples were pooled and sequenced on the Illumina MiSeq using the MiSeq^®^ (Illumina, San Diego, CA, USA) Reagent Kit v3 600 cycles sequencing chemistry. The library was clustered to a density of approximately 1200 K/mm2.

### 3.8. Genome Assembly

The quality control of reads was checked with FASTQC software [43] to evaluate the GC%, number of k-mers, sequence length, and total reads. Trimmomatic v0.36 [44] was used to filter low quality sequences and adapters. Filtered reads were assembled with SPAdes v3.11.0 [45] using default k-mer lengths. The obtained contigs were evaluated with QUAST tool [46] to select the best quality contig. Finally, Prokka [47] was used to annotate the draft genome. 

### 3.9. Secondary Metabolites Gene Clusters Search

The online platform of Antismash [30] was used to detect the secondary metabolites gene clusters present in the draft genome. 

### 3.10. Antibiotic Activity Test

To test the antibiotic activity, we used the disc diffusion method [48] as a primary indicator. Thus, compound (**1**) and (**2**) were tested to determine their activity on *Staphylococcus lentus* DSM 20352^T^, and *Escherichia coli* DSM 498^T^. These bacteria were cultured in GYM medium (4 g glucose, 4 g yeast extract, 10 g malt extract, 2 g CaCO_3_, 1 L deionized water, pH = 7.2, and 12 g agar). Lupinacidin A (**1**), and Galvaquinone B (**2**) were transferred to a paper disc to reach a final concentration of 25 μg and 50 μg each in triplicate. Additionally, we used an antibiotic susceptibility disc of streptomycin (Oxoid^®^, Columbia, MD, USA) as a positive indicator of antibiotic activity. The plates were inoculated with fresh culture of *Staphylococcus lentus* DSM 20352^T^, and *Escherichia coli* DSM 498^T^, and incubated at 37 °C for 24 h. After the incubation period, the inhibition zone was measured and registered.

## 4. Conclusions

We established that the Easter Island sea anemone *Gyractis sesere* contained two anthraquinones, Lupinacidin A (**1**) and Galvaquinone B (**2**), which were ultimately found to be produced by one of the Actinobacteria associated with this marine invertebrate, *Verrucosispora* sp. SN26_14.1. The production of the identified metabolites by the bacterial isolate apparently follows a recently characterized PKS type II pathway with a Baeyer−Villiger type rearrangement assembly line. Our finding adds a new actinobacterial genus to the producers of these anthraquinones, implying that these metabolites are not exclusive to the genera *Streptomyces* and *Micromonospora*. It was demonstrated, that culture-based approaches remain as effective tools for the isolation of polyketide producing Actinobacteria as sources for secondary metabolites of potential use in drug discovery. Our study confirms that cnidarians, and in specific sea anemones, can be a source of such pharmacologically relevant microorganisms. Finally, these findings re-open the debate about the real producers of secondary metabolites in sea animals and add another example of associated bacteria as producers of substances present in sea animals. In addition, the study provides information on the chemistry harbored in biota of the geographically isolated and almost unstudied, Easter Island. 

## Figures and Tables

**Table 1:** Spectroscopic NMR data of Lupinacidin A (**1**) and Galvaquinone B (**2**).

		Lupinacidin A				Galvaquinone B		
Position	δ_C_	δ_H_ Mult (J in Hz)	HMBC	COSY	δ_C_	δ_H_ Mult (J in Hz)	HMBC	COSY
1	162.5				157.5			
2	117.5				137.1			
3	159.6				141.1			
4	130.4				153.8			
4a	127.9				116.2			
5	190.2				190.7			
5a	117.1				116.2			
6	162.6				162.7			
7	124.3	7.26, d (8.2)	5a, 6, 9	H-8	124.8	7.32, d (8.5, 1.3)	5a, 6, 9	H-8
8	136	7.62, dd (8.2, 7.5)	6, 9a	H-7, H-9	137.1	7.72, dd (8.5, 7.6)	6, 9, 9a	H-7, H-9
9	118.3	7.79, d (7.5)	5a, 7, 10	H-8	119.7	7.90, d (7.6, 1.3)	5a, 7, 10,	H-9
9a	133				133.4			
10	186.9				186.5			
10a	110.8				111.7			
11	8.4	2.27, s	1, 2, 3		13.2	2.25, s	1, 2, 3	
12	24.8	3.21, m (6.6)	3, 4a, 13, 14	H-13	204.9			
13	37.7	1.46, m (6.6)	4a, 12, 14, 15	H-12, H-14	42.4	2.85, m	12, 14, 15	H-14
14	28.4	1.80, m (6.6)	12, 13, 15, 16	H-13, H-15, H-16	31.9	1.63, m	14, 15, 16, 17	H-15
15	22.5	1.04, d (6.6)	13, 14, 16	H-14	27.6	1.63, m	14, 15, 16, 17	H-13, H-16, H-17
16	22.5	1.04, d (6.6)	13, 14, 15	H-14	22.4	0.93, d, (6.2)	14, 15, 17	H-15
17					22.4	0.93, d, (6.2)	14, 15, 16	H-15
1-OH		14.18	1, 2, 10a,			13.49, s	1, 2, 10a	
3-OH		5.62	2, 4a, 3					
4-OH						12.50, s	3, 4, 10a	
6-OH		12.96	5a, 6, 7			12.14, s	5a, 6, 8	

** ^1^H NMR (600 MHz) Solvent: CDCl_3_ (δ^1^H, mult, J in Hz), *** ^13^C NMR (125 MHz), Solvent: CDCl_3._

## Supplementary Materials

The following are available online at https://www.mdpi.com/1660-3397/17/3/154/s1. Information on NMR spectra, HRLCMS data, and secondary metabolite gene cluster.
