# Supplementary material for: Antitumor Anthraquinones from an Easter Island Sea Anemone: Animal or Bacterial Origin?
Source: Mar Drugs. 2019 Mar 5;17(3):154. doi: 10.3390/md17030154 (PMC6471592; doi:10.3390/md17030154)
Supplement: Supplementary file 1 [file marinedrugs-17-00154-s001.pdf]

Antitumor anthraquinones from an Easter Island sea anemone: Animal or bacterial origin?

Ignacio Sottorff[1,2], Sven Künzel[3], Jutta Wiese[1], Matthias Lipfert[4], Nils Preußke[4], Frank D. Sönnichsen[4], and Johannes F. Imhoff[1,*]

[1] GEOMAR Helmholtz Centre for Ocean Research Kiel, Marine Microbiology, Kiel, Germany; isottorff@geomar.de, jwiese@geomar.de, jimhoff@geomar.de
[2] Facultad de Ciencias Naturales y Oceanográficas, Universidad de Concepción, Chile.
[3] Max Planck Institute for Evolutionary Biology, Plön, Germany; kuenzel@evolbio.mpg.de
[4] Otto Diels Institute for Organic Chemistry, University of Kiel; fsoennichsen@oc.uni-kiel.de, mlipfert@oc.uni-kiel.de, npreusske@oc.uni-kiel.de

* **Correspondence:** jimhoff@geomar.de; Tel.: +49 431 600-4450

Supplementary Data Index.

Suppl. Table I. Isolated actinobacterial symbionts from the Easter Island sea anemone *Gyractis sesere*……………………………...4

Suppl. Fig. 1. BPC extraction of sea anemone chloroform extract for lupinacidin A (MW: 340)…………………………………....5

Suppl. Fig. 2. BPC extraction of sea anemone chloroform extract for galvaquinone B (MW: 368)…………………………….…...6

Suppl. Fig. 3. BPC extraction of Lupinacidin A **(1)** (MW: 340) from the crude extract of *Verrucosispora* sp. SN26_14.1 isolated from the Easter Island sea anemone, *Gyractis sesere*..………………………………………………………………………………..7

Suppl. Fig. 4. BPC extraction of Galvaquinone B **(2)** (MW: 368) from the crude extract of *Verrucosispora* sp. SN26_14.1 isolated from the Easter Island sea anemone, *Gyractis sesere*..………………………………………………………………………….8

Suppl. Fig. 5. Fraction 2 chromatogram of the crude extract of *Verrucosispora* sp. SN26_14.1, showing the elution of the Lupinacidin A **(1)** and Galvaquinone B **(2)** ……………………………………………………………………………………..…9

Suppl. Fig. 6. Purified Lupinacidin A **(1)** and Galvaquinone B **(2)** obtained from the sea anemone symbiont, *Verrucosispora* sp. SN26_14.1……10

Suppl. Fig. 7. UV profile for Lupinacidin A **(1)**. ……………………………………………………………………………11

Suppl. Fig. 8. UV profile for Galvaquinone B **(2)**. …………………………………………………………………………12

Suppl. Fig. 9. HRLCMS for Lupinacidin A **(1)**………………………………………………………..……………...13

Suppl. Fig. 10. HRLCMS for Galvaquinone B **(2)**……………………………………………………………………14

Suppl. Fig. 11. ^{1}H Lupinacidin A **(1)**……………………………………………………………………………...…15

Suppl. Fig. 12. ^{13}C Lupinacidin A **(1)**…………………………………………………………………….………16

Suppl. Fig. 13. HSQC for Lupinacidin A **(1)**……………………………...…………………………....17

Suppl. Fig. 14. HMBC for Lupinacidin A **(1)**……………………………………………………………..…18

Suppl. Fig. 15. COSY for Lupinacidin A **(1)**………………………………………………………….….…19

Suppl. Fig. 16. ^{1}H Galvaquinone B **(2)**………………………………………………………….……...20

<u>Suppl. Fig. 17.</u> ^{13}C Galvaquinone B **(2)**…………………………………………………………………………………..…..21
<u>Suppl. Fig. 18.</u> HSQC Galvaquinone B **(2)**……………………………………………………………………………..…....22
<u>Suppl. Fig. 19.</u> HMBC Galvaquinone B **(2)**………………………………………………………………………….......… 23
<u>Suppl. Fig. 20.</u> COSY Galvaquinone B **(2)**…………………………………………………………………………………..24
<u>Suppl. Fig. 21.</u> Rsd, Rsl, and Vex gene clusters comparison of the Lupinacidin A **(1)** and Galvaquinone B **(2)** producers………….25
<u>Suppl. Fig. 22.</u> Complementary evaluation of the sea anemone crude extract…………………………………………………………….26

Table I. Isolated actinobacterial symbionts from the Easter Island sea anemone *Gyractis sesere*.

Media	Sample	Closest type strain obtained from BLAST	Sequence length	Access number NCBI	% identity
BSEM	SN26_101.1	*Cellulosimicrobium funkei* DSM 16025 ^{T}	1476	MH299430	99
BCM	SN26_23.1	*Arthrobacter koreensis* JCM 12361^{T}	1338	MH299428	99
BCM	SN26_6.1	*Arthrobacter arilaitensis* DSM 16368 ^{T}	1481	MH299429	98
BTM	SN26_12.1	*Rhodococcus soli DSM 46662*^{T}	1475	MH299445	99
BCM	SN26_22.1	*Streptomyces drozdowiczii* JCM 13580 ^{T}	1336	MH299485	99
BTM	SN26_13A.1	*Dietzia cinnamea* DSM 44904 ^{T}	1471	MH299435	99
BTM	SN26_14.1	*Verrucosispora maris* DSM 45365 ^{T}	1538	MK332504	99
BTM	SN26_25.1	*Micromonospora rifamycinica DSM 44983* ^{T}	1417	MH299468	99
SIMA1	SN26_100.1	*Micromonospora purpureochromogenes DSM 43821* ^{T}	1364	MH299466	98
BTM	SN26_110.1	*Micromonospora echinospora DSM 43816* ^{T}	1330	MH299467	99

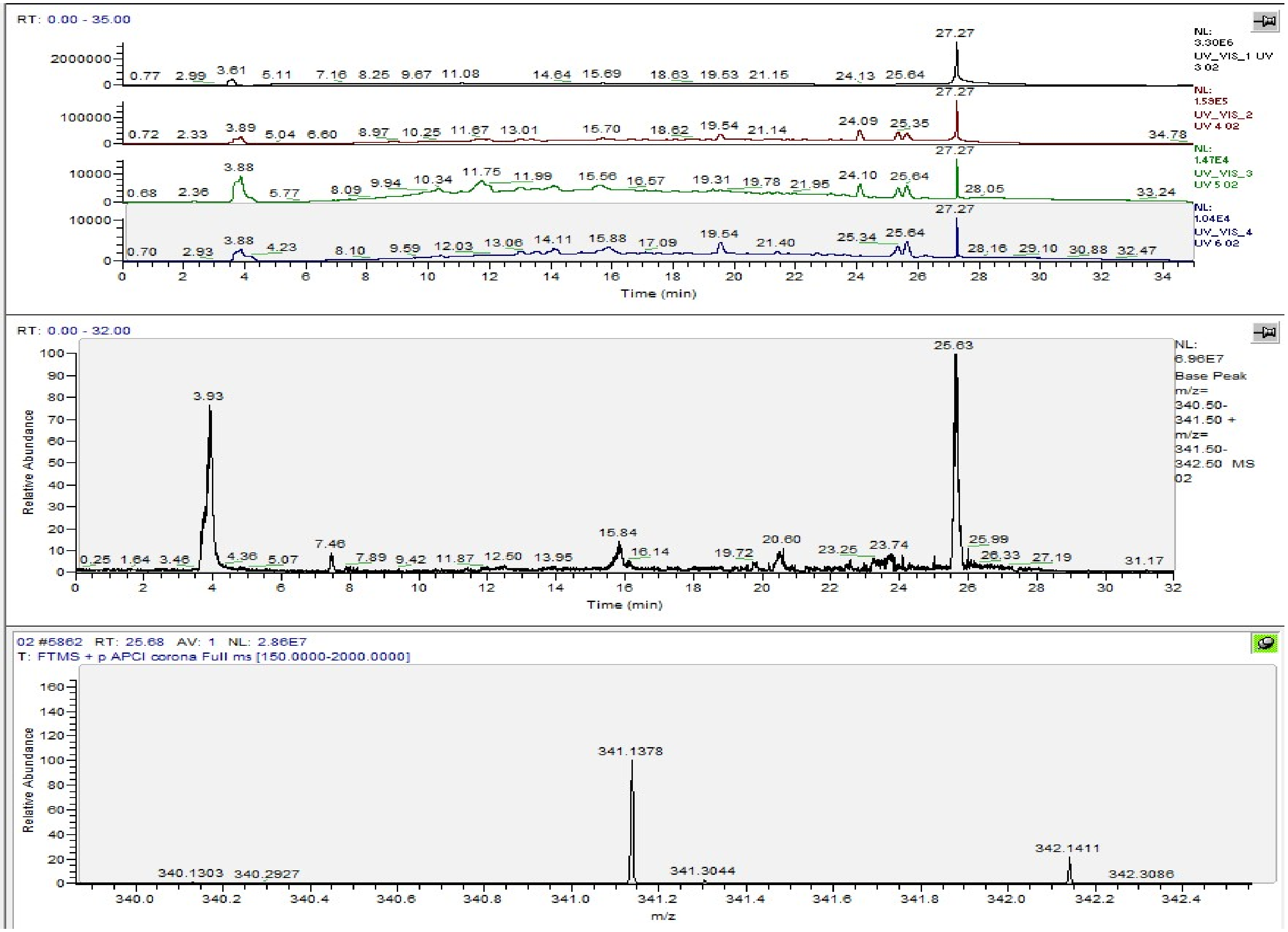

Suppl. Fig. 1. BPC extraction of Sea anemone chloroform extract for Lupinacidin A **(1)** (MW: 340).

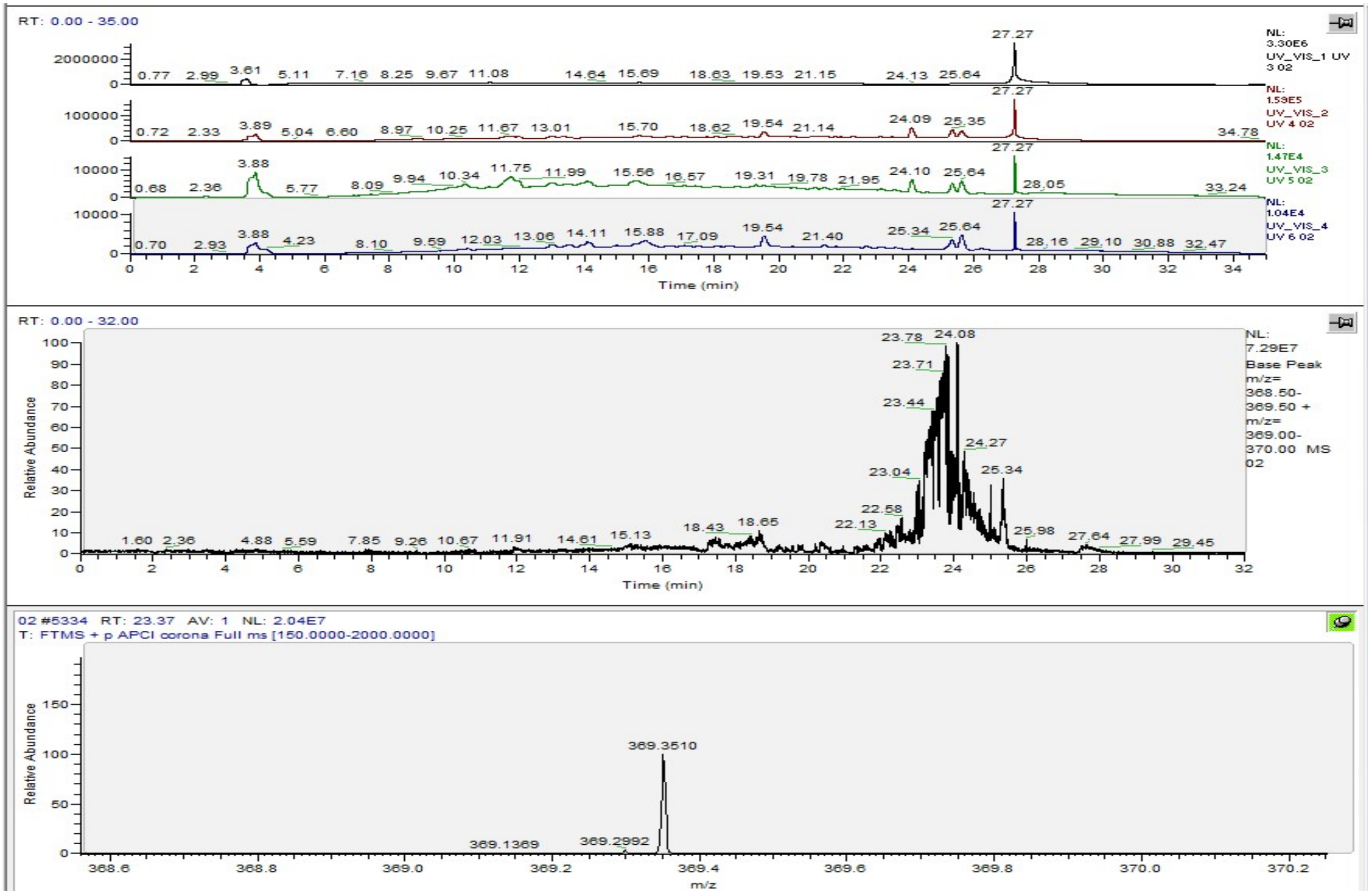

Suppl. Fig. 2. BPC extraction of Sea anemone- Chloroform extract for Galvaquinone B **(2)** (MW: 368).

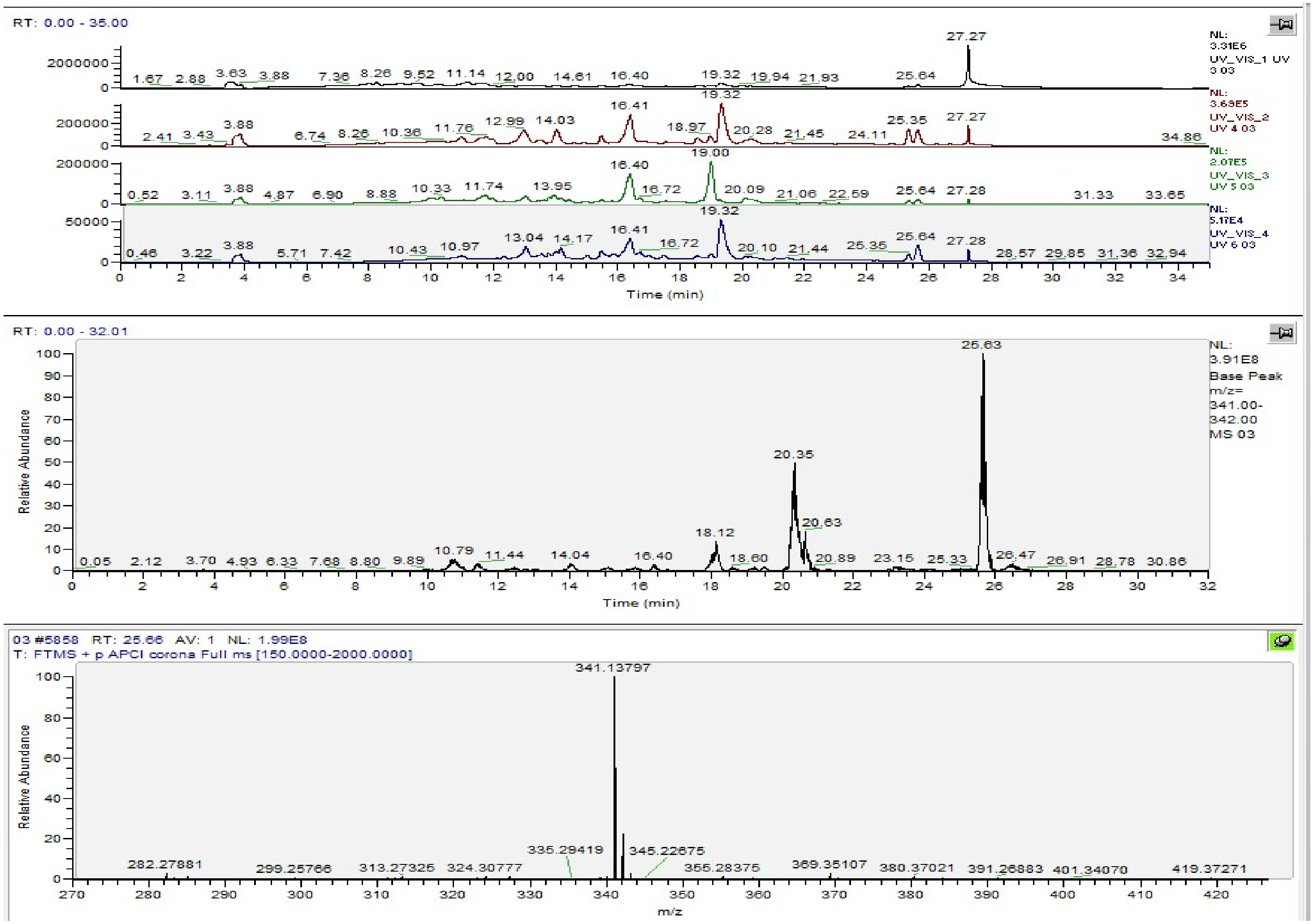

Suppl. Fig. 3. BPC extraction of Lupinacidin A (**1**) (MW: 340) from the crude extract of *Verrucosispora* sp. SN26_14.1 isolated from the Easter Island sea anemone, *Gyractis sesere.*

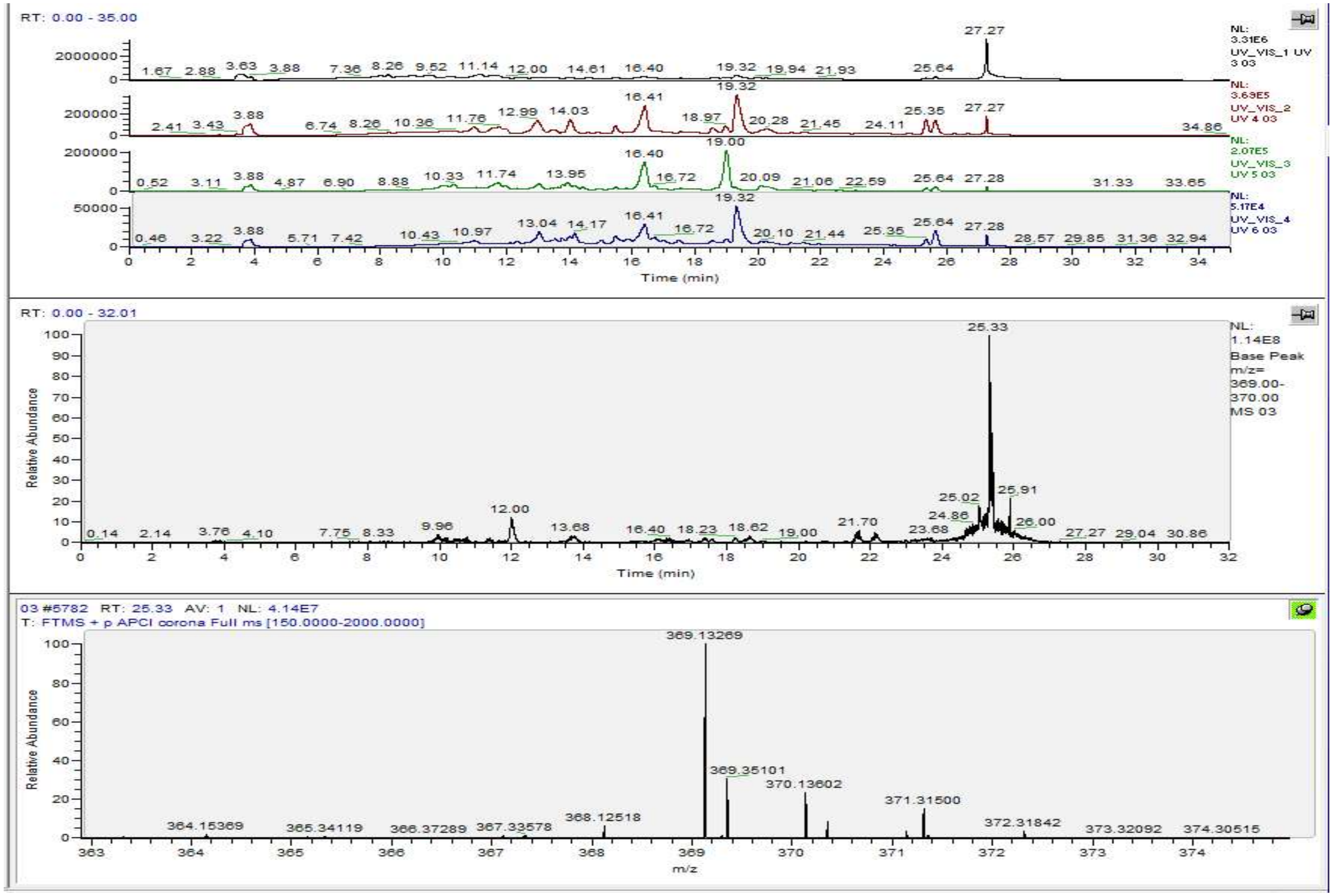

Suppl. Fig. 4. BPC extraction of Galvaquinone B (**2**) (MW: 368) from the crude extract of *Verrucosispora* sp. SN26_14.1 isolated from the Easter Island sea anemone, *Gyractis sesere.*

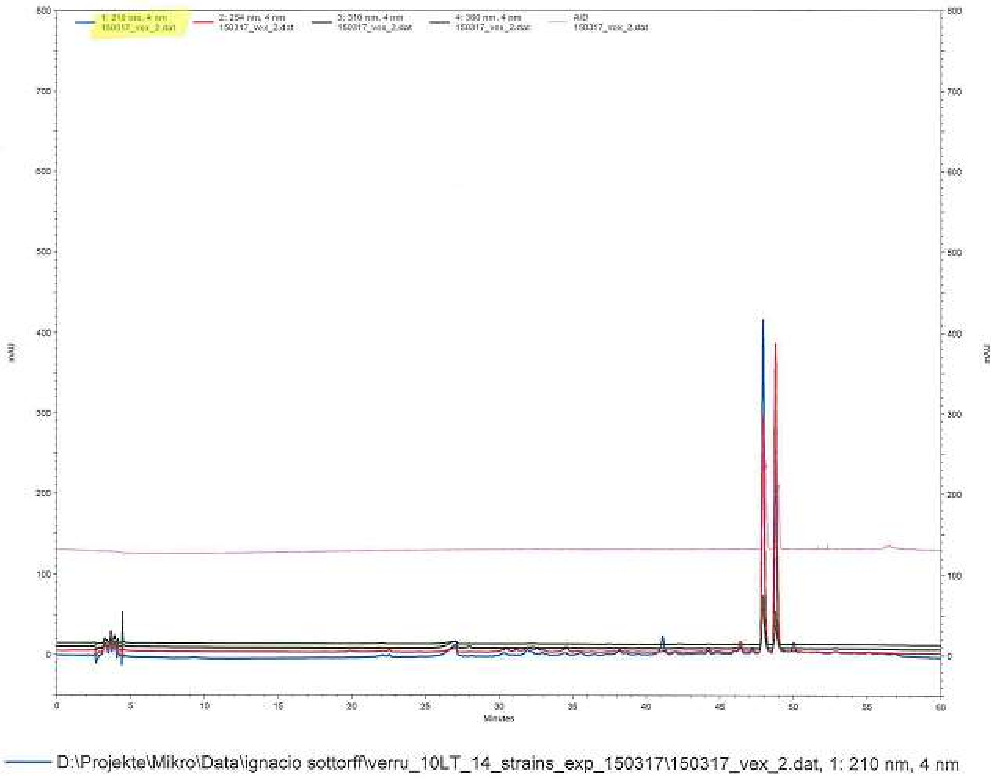

Suppl. Fig. 5. Fraction 2 Chromatogram of the crude extract of *Verrucosispora sp.* SN26_14.1, showing the elution of the Lupinacidin A (**1**) and Galvaquinone B (**2**).

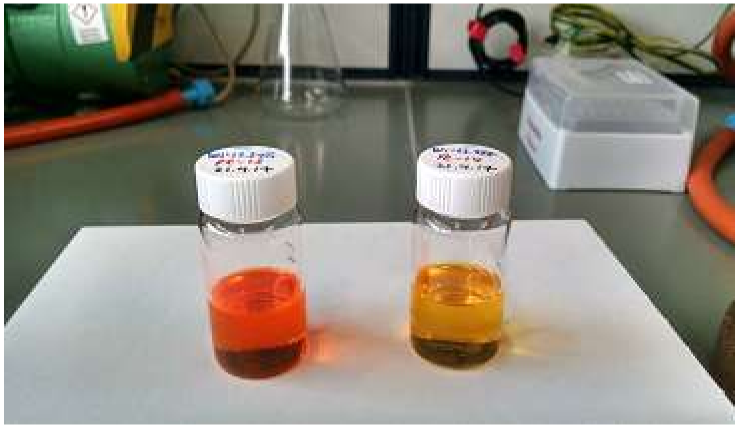

<u>Suppl. Fig. 6.</u> Purified Galvaquinone B (**2**) (left) and Lupinacidin A (**1**) (right) obtained from the sea anemone symbiont, *Verrucosispora* sp. SN26_14.1.

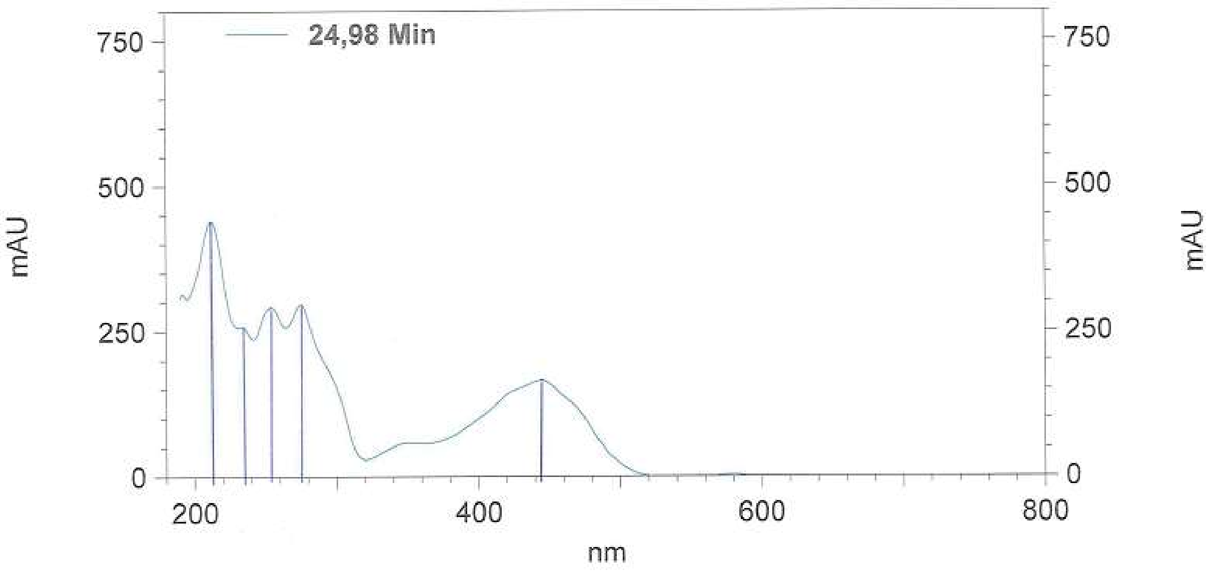

Suppl. Fig. 7. UV profile for Lupinacidin A (**1**).

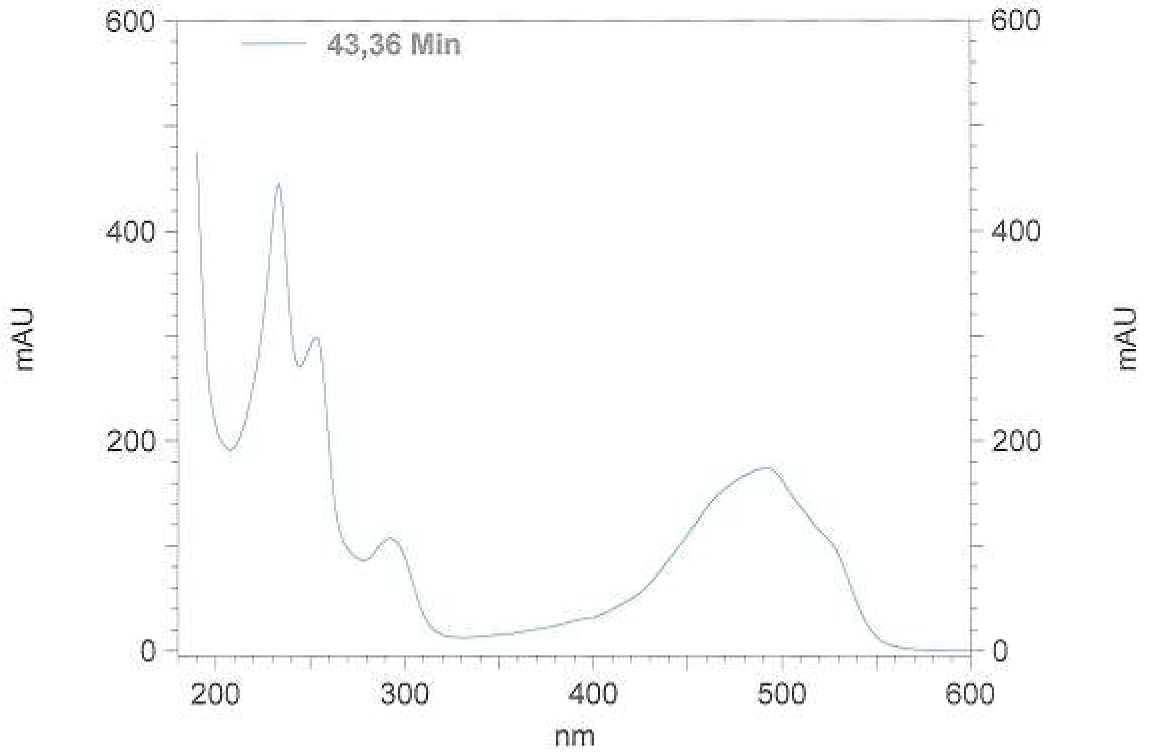

<u>Suppl. Fig. 8.</u> UV profile for Galvaquinone B (**2**).

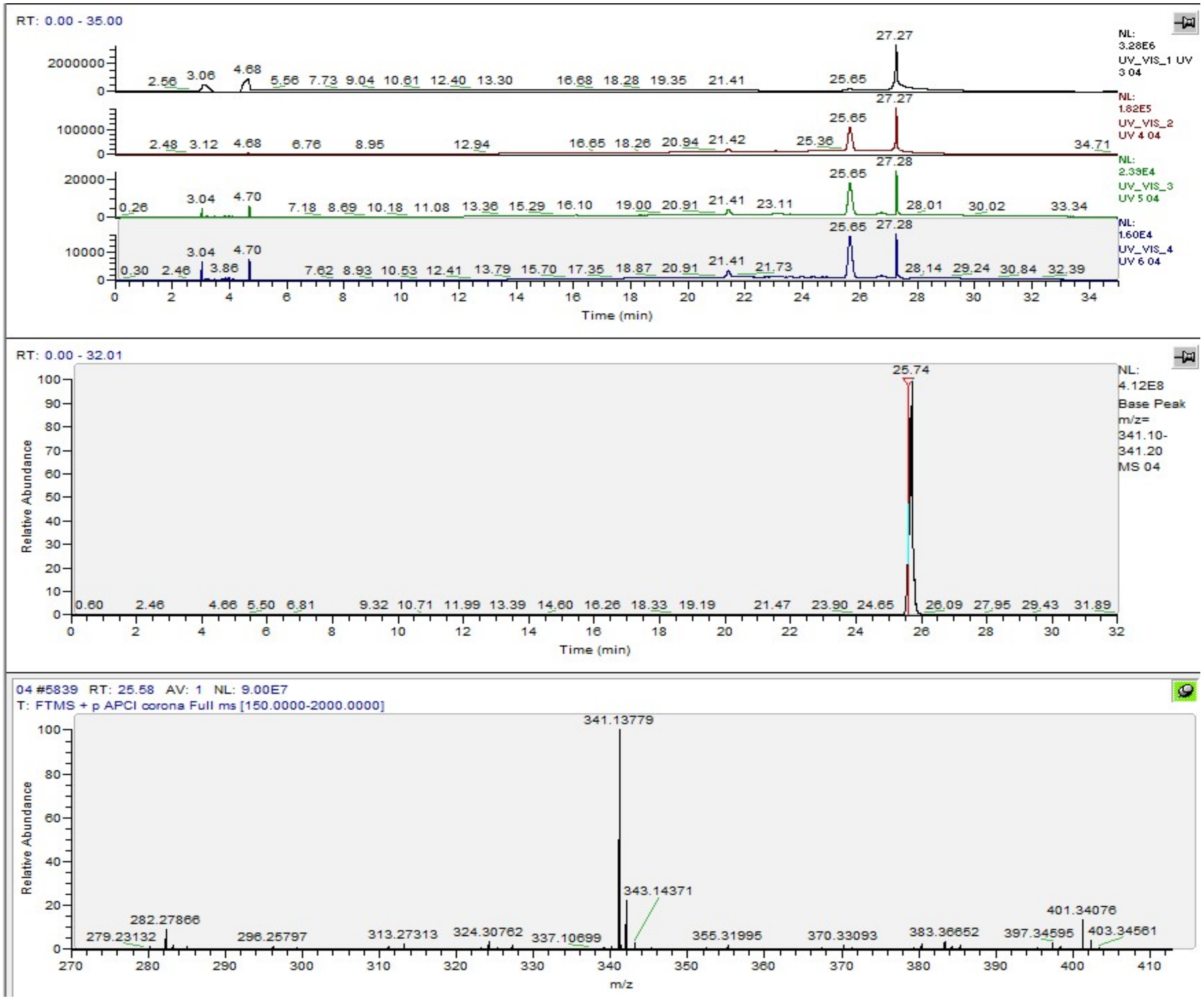

<u>Suppl. Fig. 9.</u> HRLCMS for Lupinacidin A (**1**).

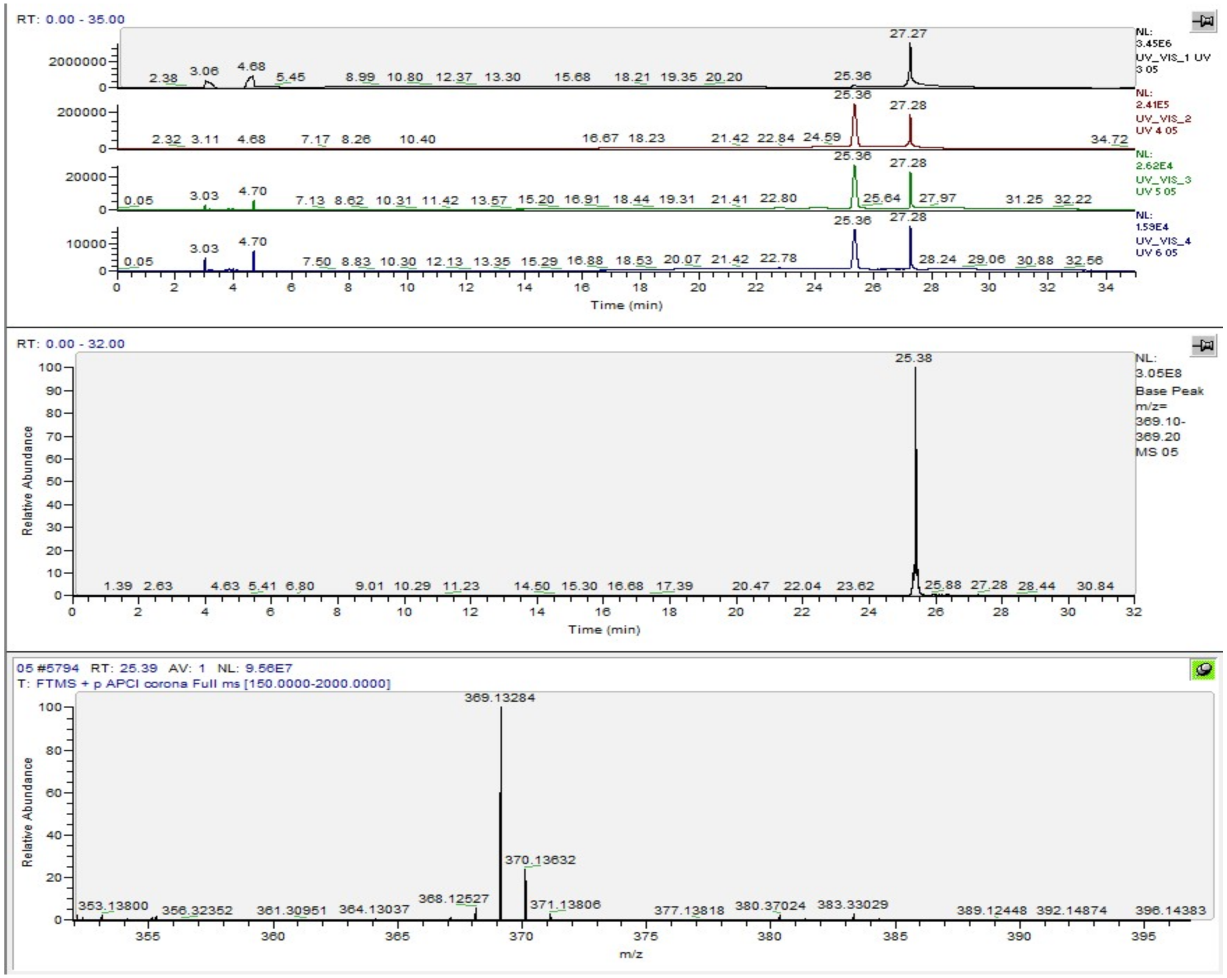

<u>Suppl. Fig. 10.</u> HRLCMS for Galvaquinone B (**2**).

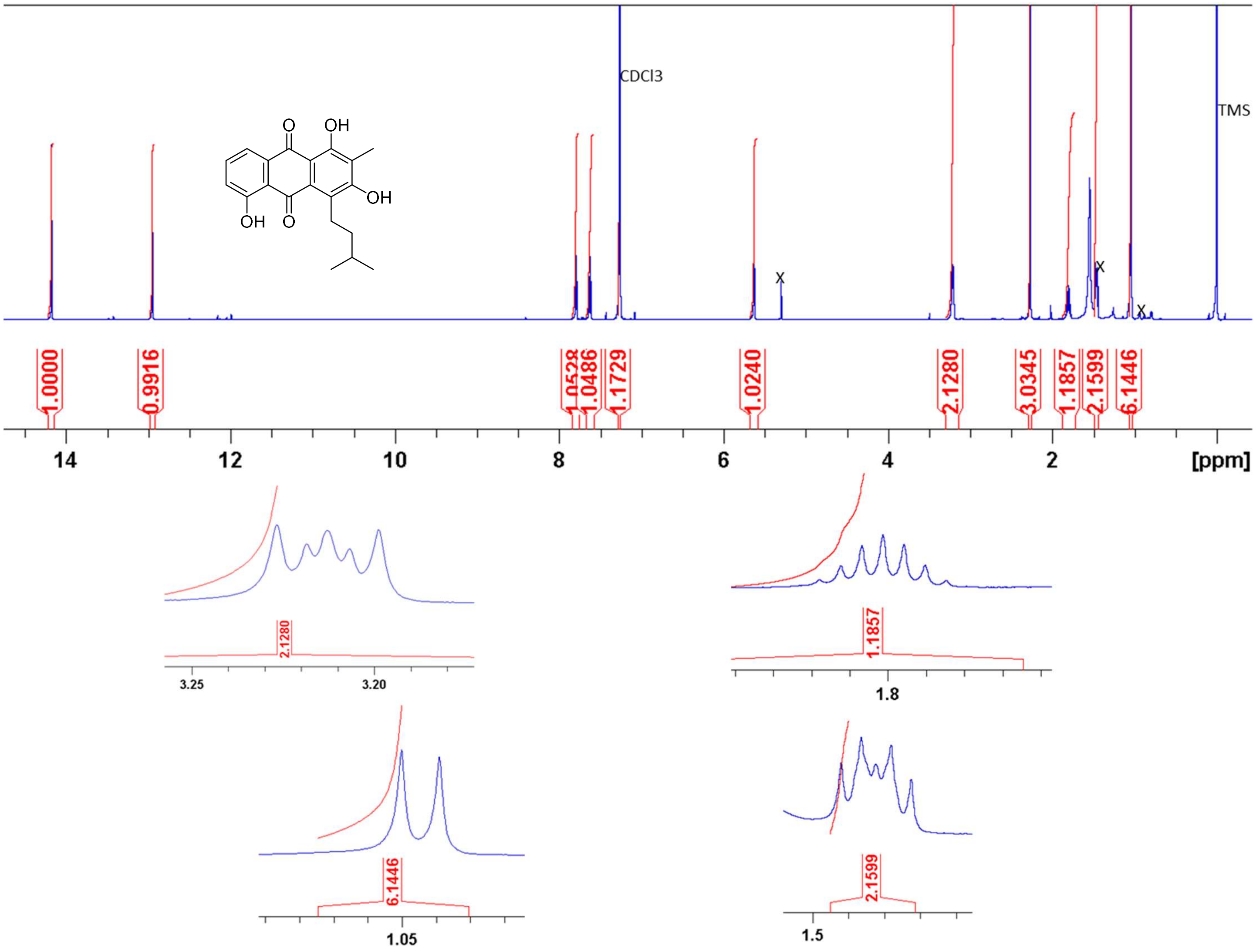

Suppl. Fig. 11. ^{1}H Lupinacidin A (**1**).

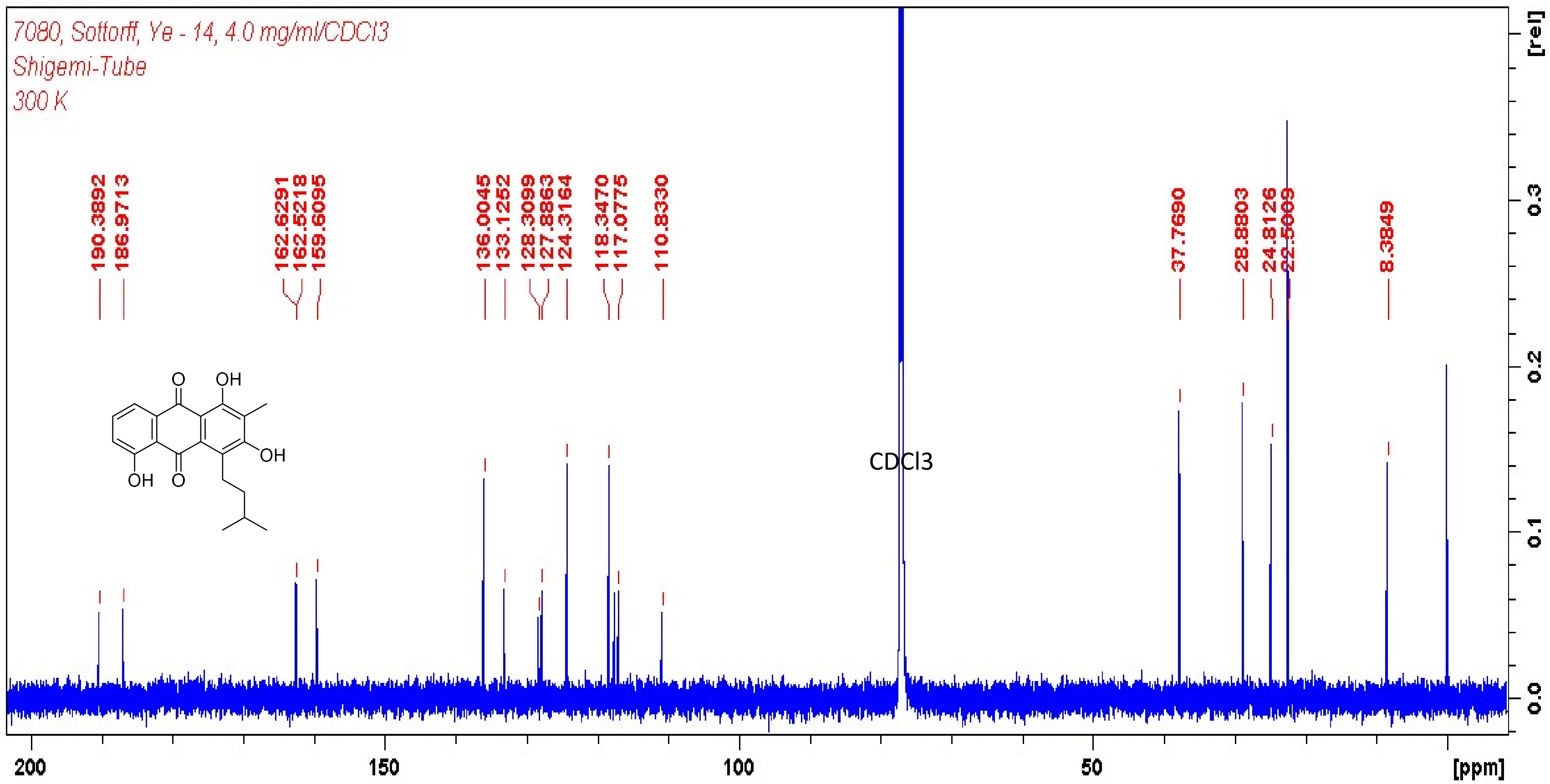

Suppl. Fig. 12. ^{13}C Lupinacidin A (**1**).

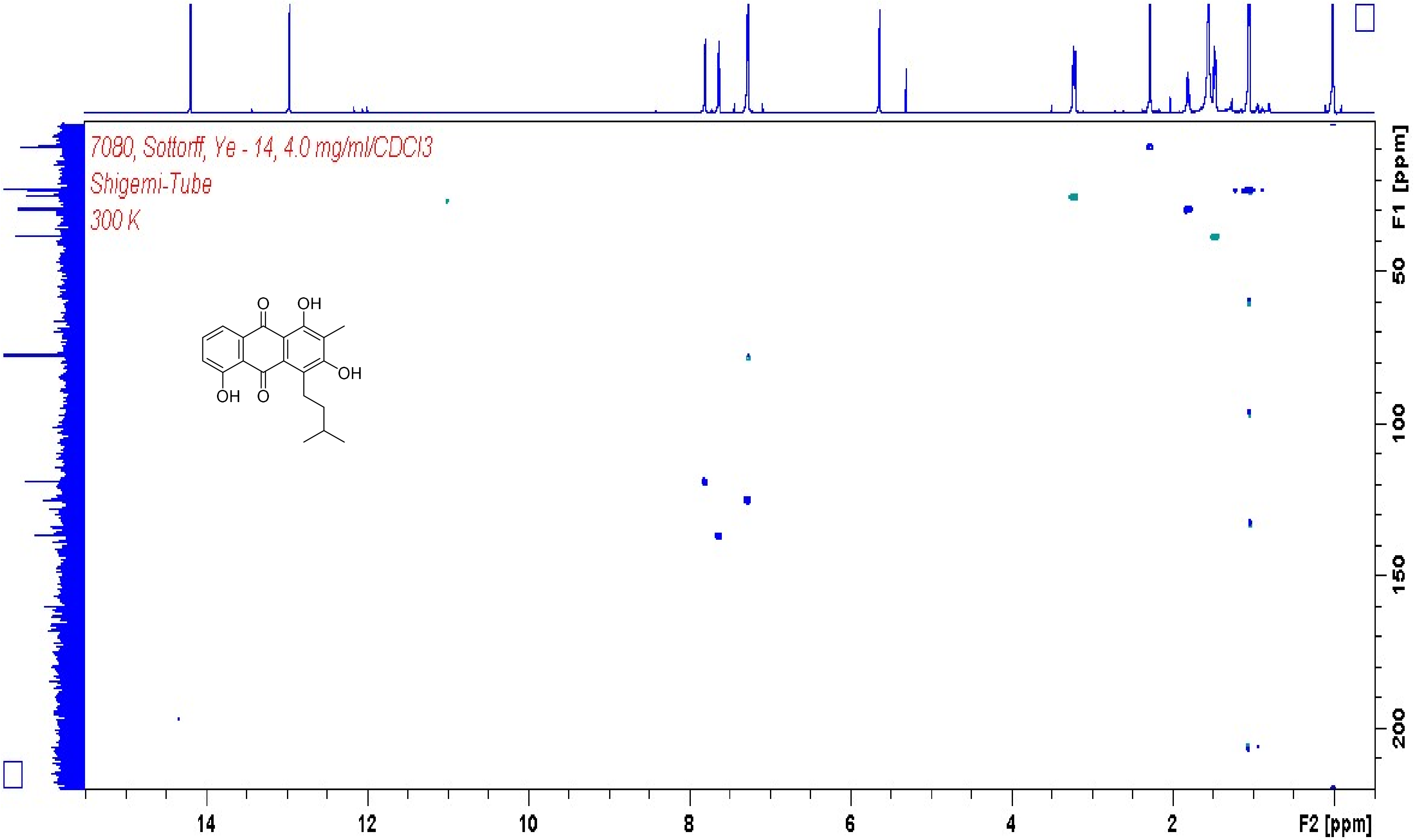

<u>Suppl. Fig. 13.</u> HSQC for Lupinacidin A (**1**).

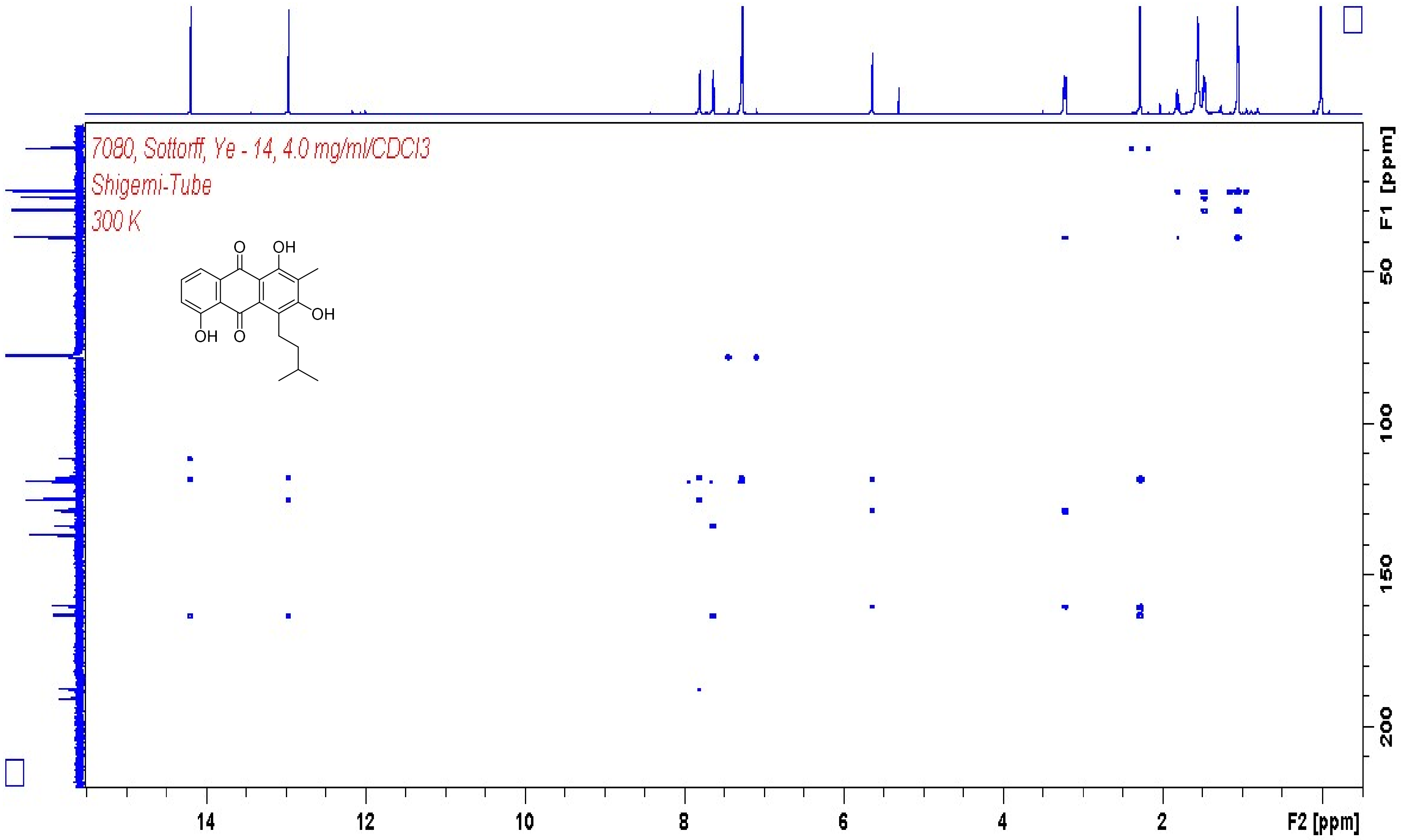

<u>Suppl. Fig. 14.</u> HMBC for Lupinacidin A (**1**).

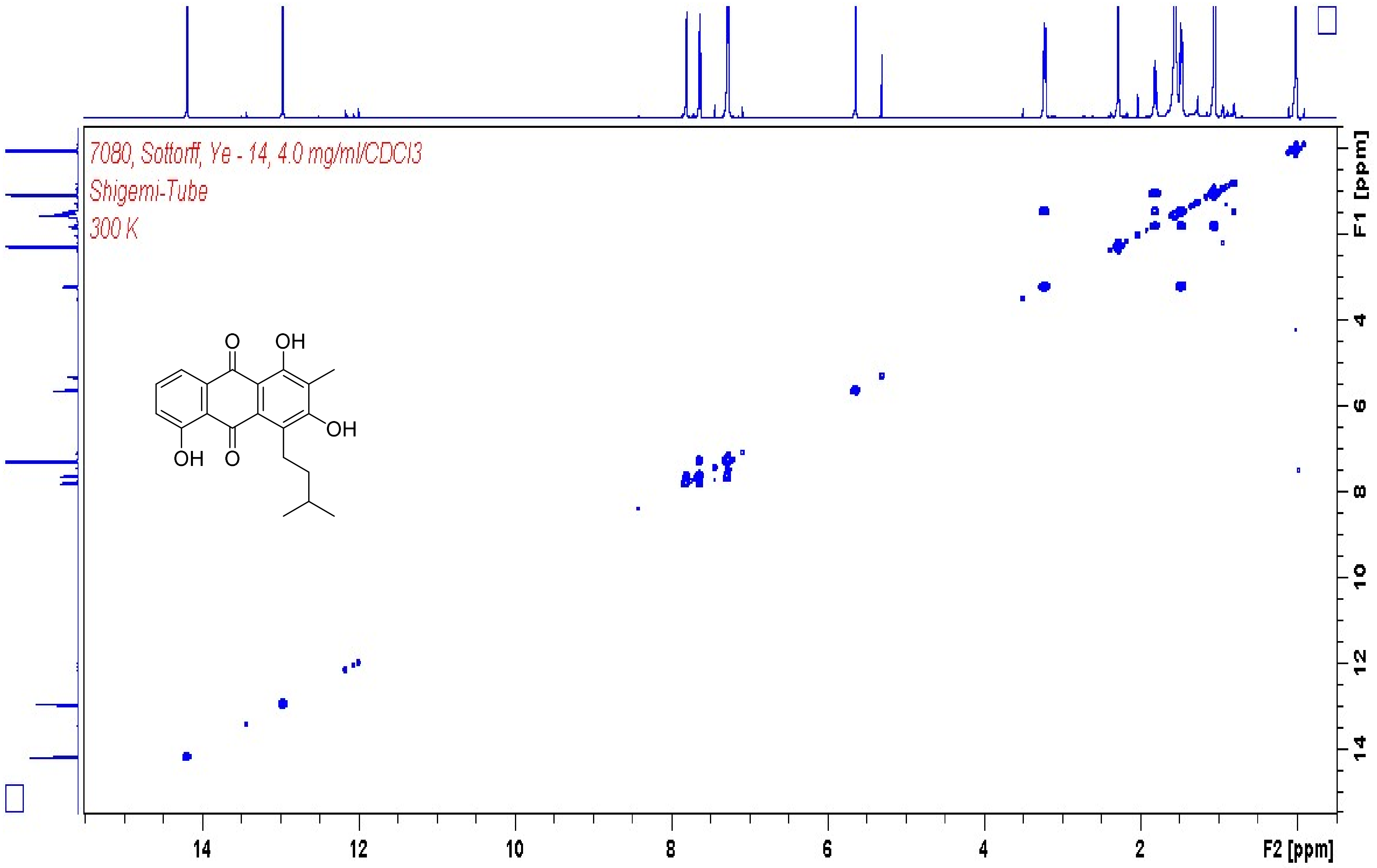

Suppl. Fig. 15. COSY for Lupinacidin A (**1**).

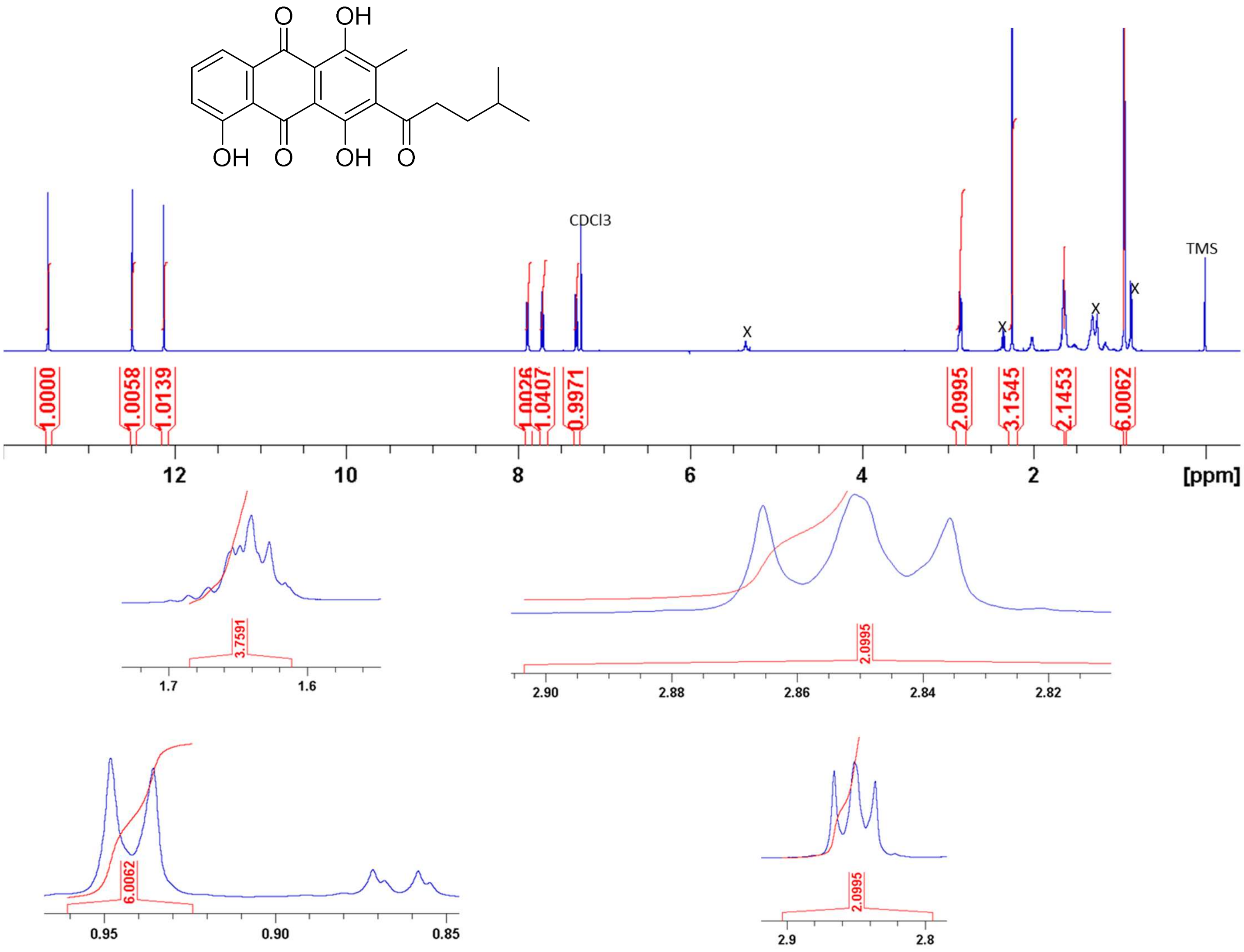

<u>Suppl. Fig. 16.</u> ^{1}H Galvaquinone B (**2**).

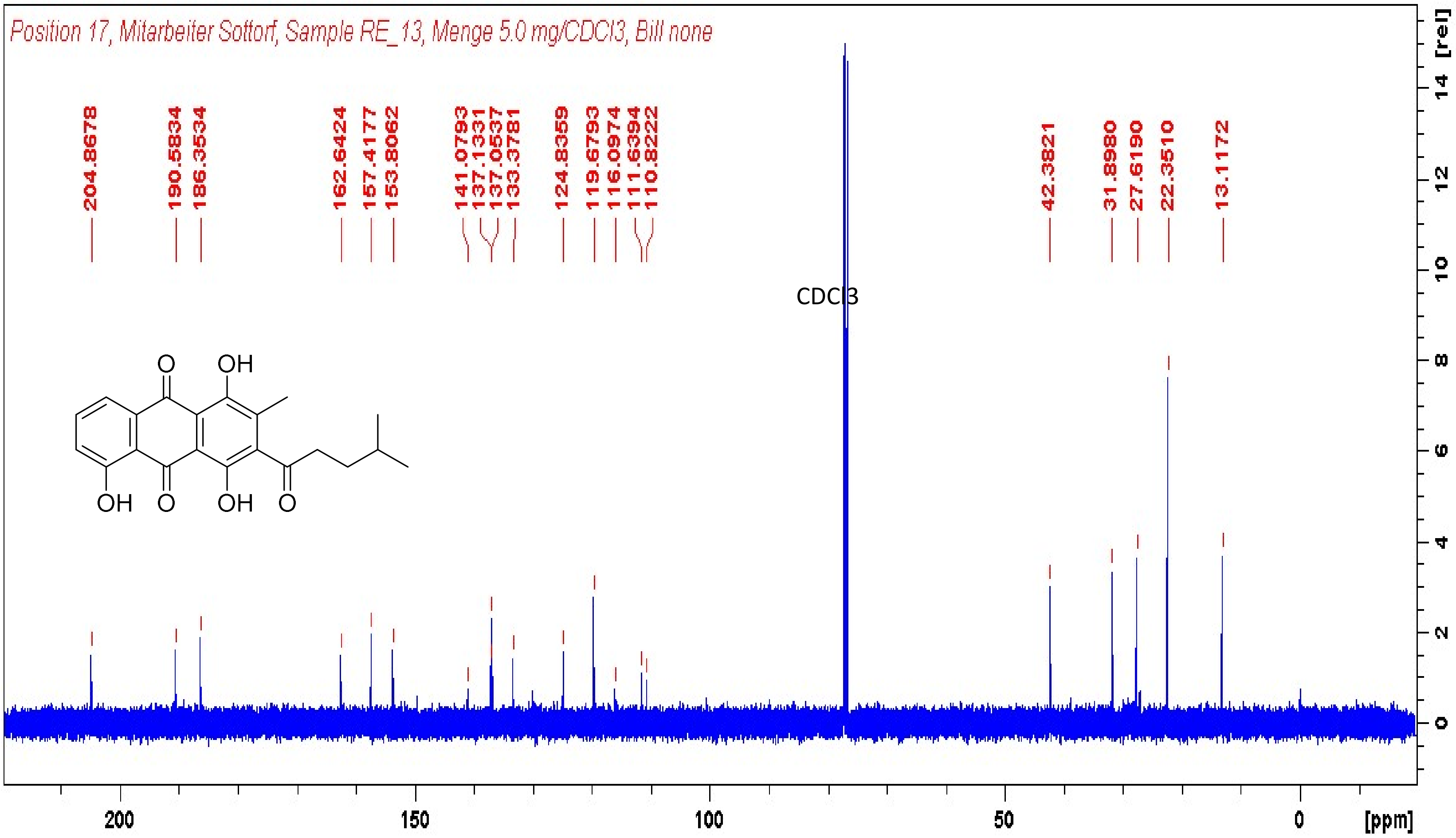

Suppl. Fig. 17. ^{13}C Galvaquinone B (**2**).

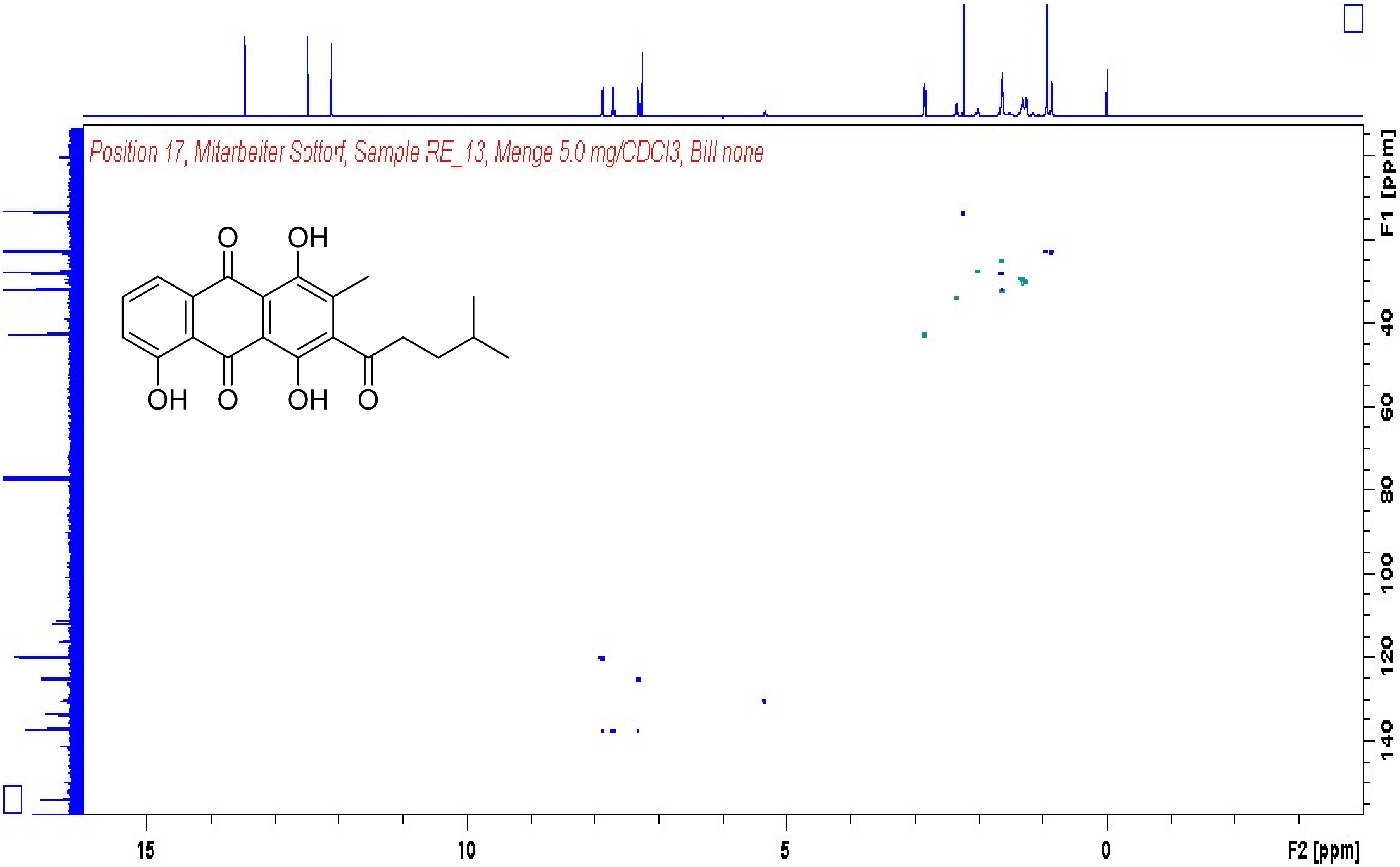

Suppl. Fig. 18. HSQC Galvaquinone B (**2**).

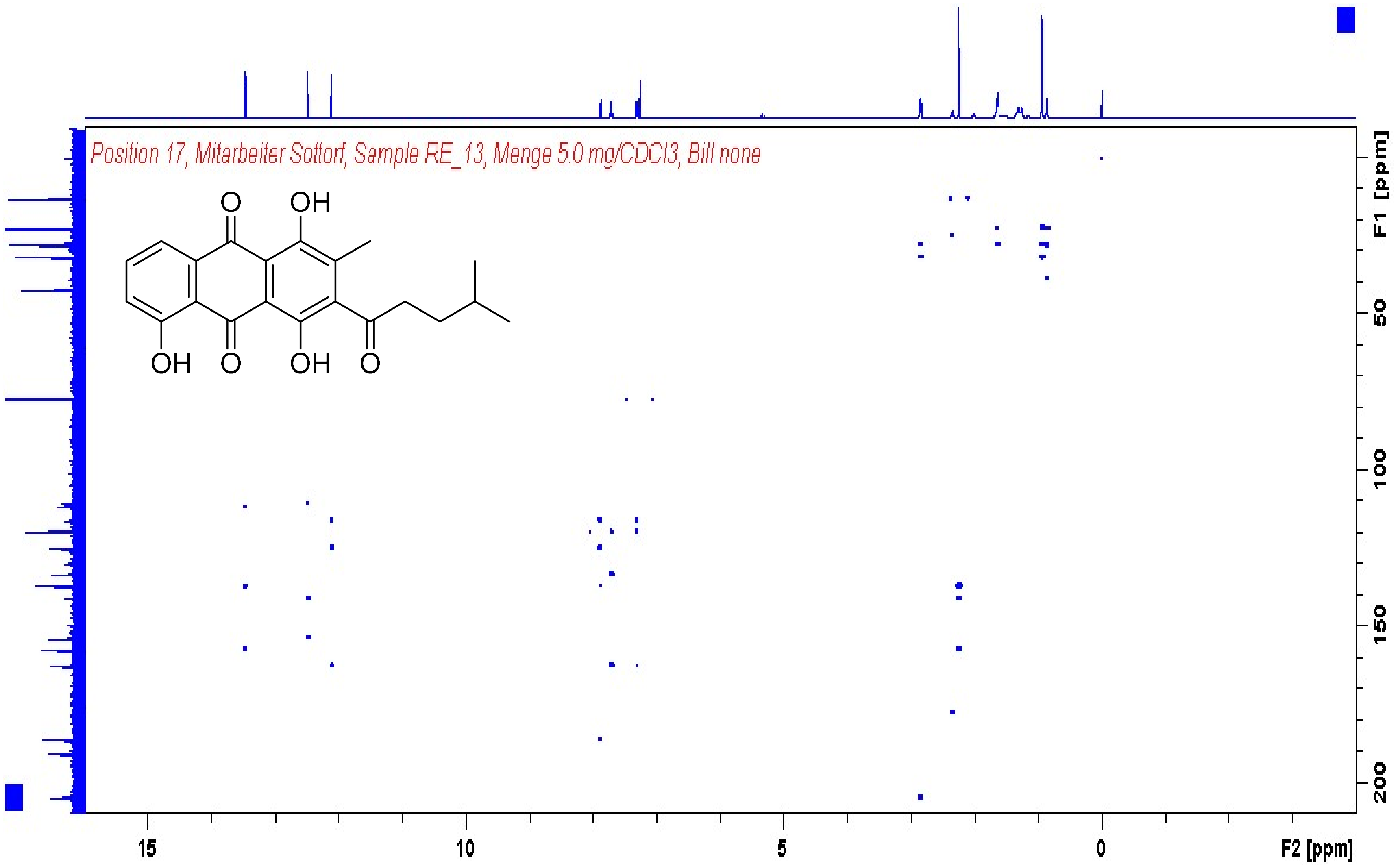

Suppl. Fig. 19. HMBC Galvaquinone B (**2**).

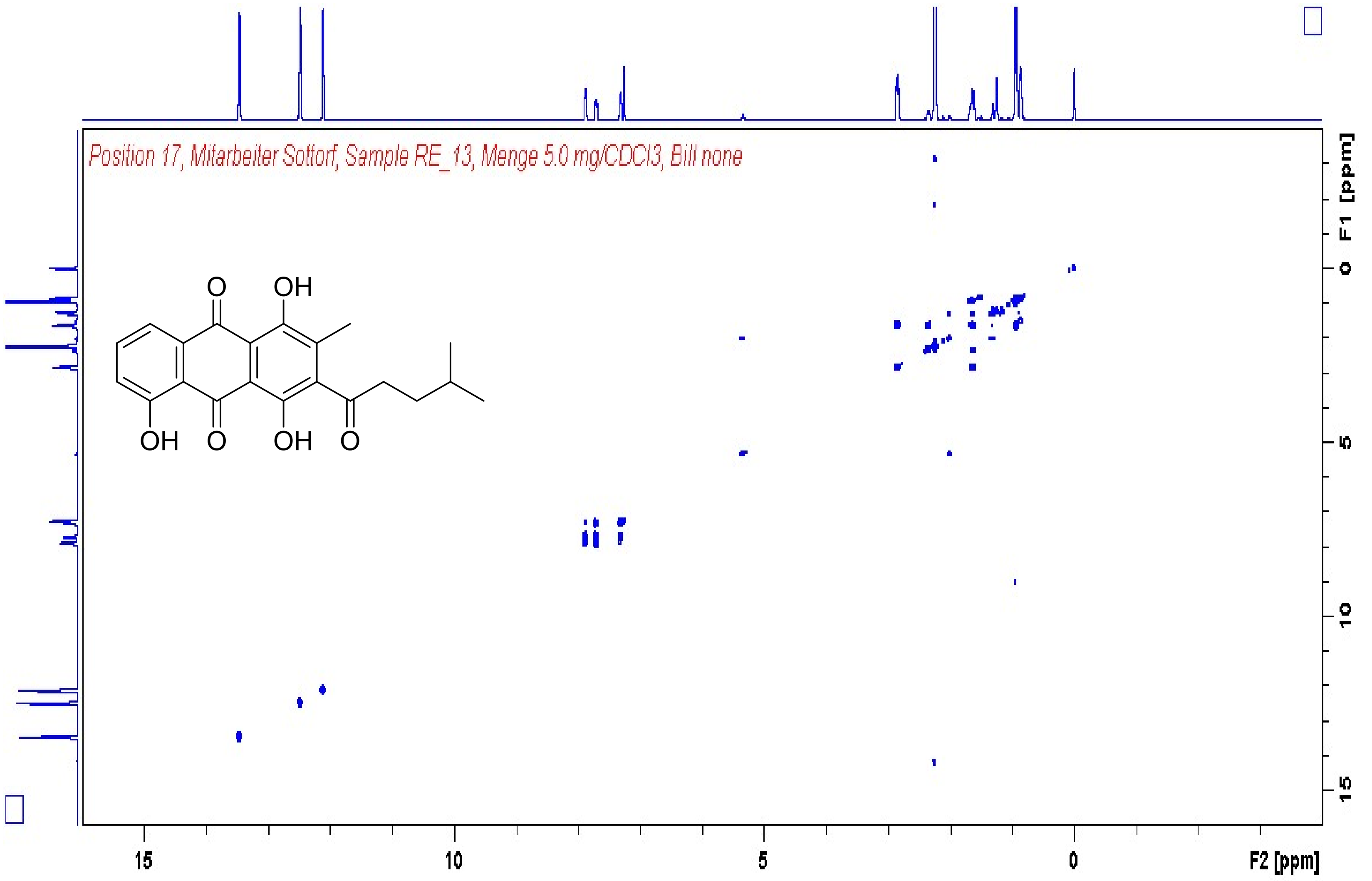

Suppl. Fig. 20. COSY Galvaquinone B (**2**).

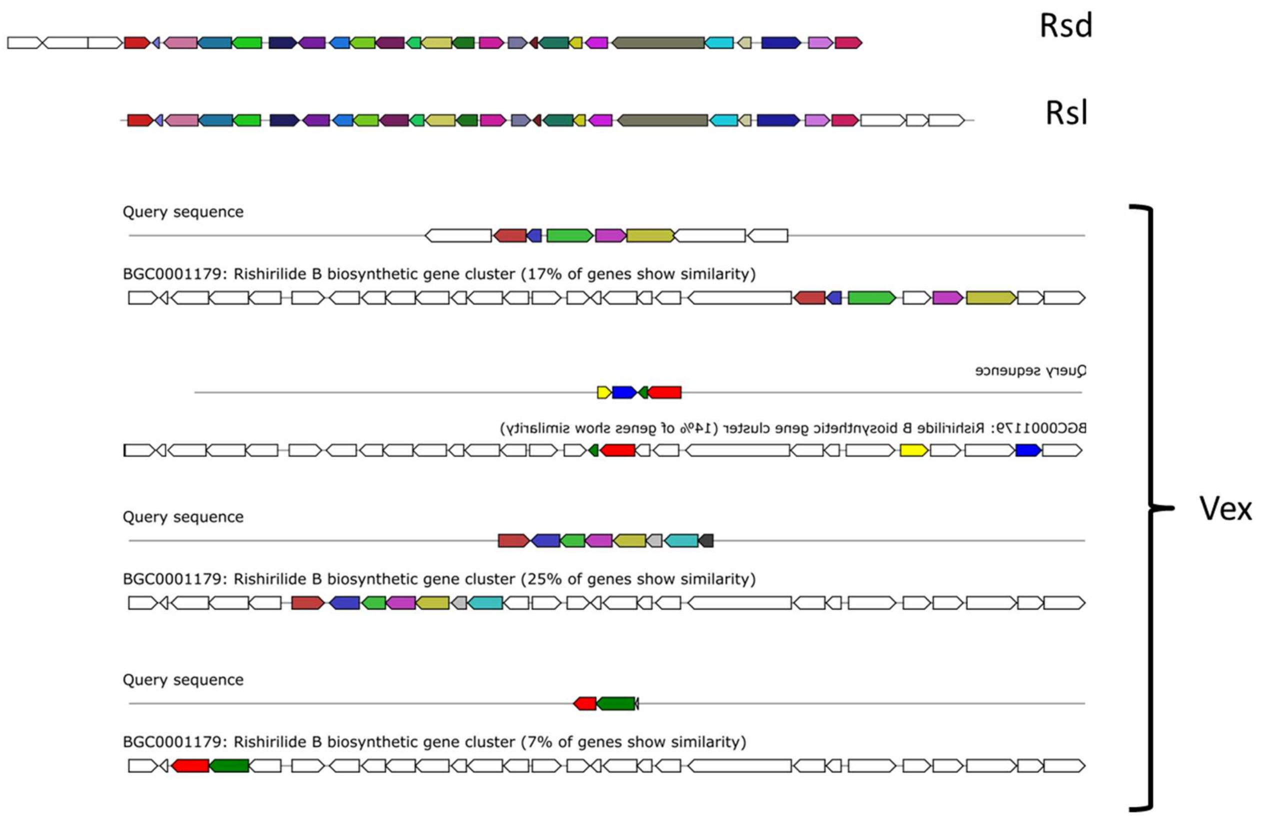

<u>**Suppl. Fig. 21.**</u> Rsd, Rsl, and Vex gene clusters comparison of the Lupinacidin A (**1**) and Galvaquinone B (**2**) producers.

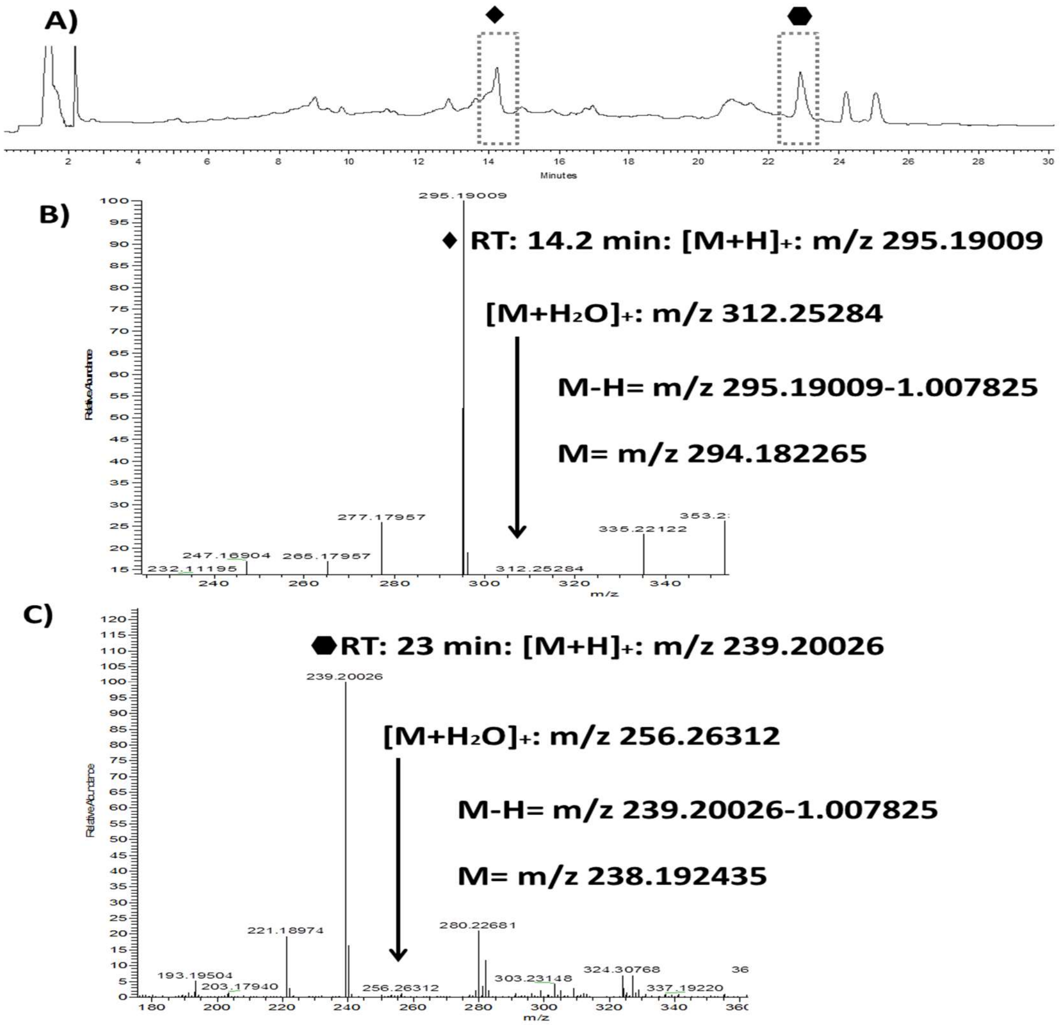

Suppl. Fig. 22. Complementary evaluation of the sea anemone crude extract. Diamond: RT:14.2 min. Hexagon: RT: 23 min. A) UV Chromatogram (254 nm) of the crude extract of the sea anemone *Gyractis sesere* highlighting the specific peaks for RT 14.2 min (diamond) and RT 23 min (hexagon). B) High resolution mass for RT 14.2 min. C) High resolution mass for RT 23 min. *RT: Retention Time.
